# Characterization of transcriptional profiles associated with stress-induced neuronal activation in Arc-GFP mice

**DOI:** 10.1038/s41380-024-02555-z

**Published:** 2024-04-22

**Authors:** Tamer Butto, Monika Chanu Chongtham, Kanak Mungikar, Dewi Hartwich, Matthias Linke, Nicolas Ruffini, Konstantin Radyushkin, Susann Schweiger, Jennifer Winter, Susanne Gerber

**Affiliations:** 1 Institute for Pharmaceutical and Biomedical Sciences, Johannes Gutenberg-University, 55128 Mainz, Germany; 2 Leibniz Institute for Resilience Research, Wallstr 7, 55122 Mainz, Germany; 3 Institute of Human Genetics, University Medical Center of the Johannes Gutenberg University Mainz, Langenbeckstr. 1, 55131 Mainz, Germany

**Keywords:** Molecular biology, Neuroscience, Psychiatric disorders

## Abstract

Chronic stress has become a predominant factor associated with a variety of psychiatric disorders, such as depression and anxiety, in both human and animal models. Although multiple studies have looked at transcriptional changes after social defeat stress, these studies primarily focus on bulk tissues, which might dilute important molecular signatures of social interaction in activated cells. In this study, we employed the Arc-GFP mouse model in conjunction with chronic social defeat (CSD) to selectively isolate activated nuclei (AN) populations in the ventral hippocampus (vHIP) and prefrontal cortex (PFC) of resilient and susceptible animals. Nuclear RNA-seq of susceptible vs. resilient populations revealed distinct transcriptional profiles linked predominantly with neuronal and synaptic regulation mechanisms. In the vHIP, susceptible AN exhibited increased expression of genes related to the cytoskeleton and synaptic organization. At the same time, resilient AN showed upregulation of cell adhesion genes and differential expression of major glutamatergic subunits. In the PFC, susceptible mice exhibited upregulation of synaptotagmins and immediate early genes (IEGs), suggesting a potentially over-amplified neuronal activity state. Our findings provide a novel view of stress-exposed neuronal activation and the molecular response mechanisms in stress-susceptible vs. resilient animals, which may have important implications for understanding mental resilience.

## Introduction

Chronic stress has been shown to significantly impact mood-related phenotypes and behavior. It has been linked to various illnesses, such as cardiovascular disease, asthma, and diabetes, as well as a variety of mood-related disorders, including anxiety, social dysfunction, and depression [1]. However, despite exposure to chronic stress, some individuals develop active stress-coping strategies, showing resilient behavior.

Studies in mice exposed to aggressive conspecifics have linked stress resilience to differential gene expression and epigenetic alterations in brain regions involved in reward processing [2–4]. Using a mouse model of stress-resilience using a paradigm of chronic social defeat (CSD) coupled with genome-wide transcriptional profiling provides a valuable opportunity to uncover the molecular properties underlying stress adaptation mechanisms [5, 6]. Such studies have identified molecular networks affected by continuous stress exposure [7], and functional transcriptional alterations of specific brain regions such as the prefrontal cortex (PFC) and ventral hippocampus (vHIP) were described [8–12]. However, while global transcriptional profiles of distinct brain regions have provided relevant data, a more targeted, cell-type-specific approach may offer a fundamentally better understanding of the cellular population involved in stress processing and the development of resilient vs. susceptible behavior.

Expression of immediate early genes (IEGs) such as *Fos* and *Arc*, follow neuronal activation and are attractive tools to determine those cells carrying behavior [13, 14]. For example, the genetic manipulation of transiently active neurons using targeted recombination in active populations (TRAP) has been used in various studies to uncover the unique molecular properties of activated neuronal populations [15–20]. This experimental setup allows the selection of active neurons using fluorescence-activated nuclei sorting (FANS). Their unique molecular properties can then be analyzed independently of surrounding inactive cells.

This study aimed to uncover the molecular properties underlying stress adaptation mechanisms using the TRAP method in a mouse model for CSD stress. By isolating activated nuclei (AN) from the vHIP and PFC followed by subsequent nuclear RNA-seq (nucRNA-seq) analysis, we could identify transcriptional profile alterations occurring in AN population from resilient and susceptible mice following CSD stress exposure. Overall, this study highlights the importance of using and understanding activated neural populations associated with susceptibility and resilience to CSD and provides valuable insights into the underlying molecular mechanisms of stress adaptation.

## Material and methods

### Animals and genotyping

Adult male mice of the genotypes Arc^*creERT2 (TG/WT)*^.R26^*CAG-Sun1-sfGFP-Myc (M/WT)*^ and Arc^*creERT2 (WT/WT)*^.R26^*CAG-Sun1-sfGFP-Myc (M/WT)*^ were used for our experiments. All mice were 7–8 weeks old at the beginning of the experiments. Animals were bred in-house (cross between TG/WT CreERT2 and M/M GFP). Acclimatization to the experimental environment was done at least 3 days before the start of any behavioral experiment. All behavioral experiments were performed in accordance with the institutional animal welfare guidelines approved by the ethical committee of the state government of Rhineland-Palatinate, Germany (G-17-1-021). See genotyping primers in Supplementary Methods.

### Behavioral experiments and sample size estimation

Behavioral tests were performed on 7–8 weeks-old Arc-GFP mice (as well as the WT-GFP mice). To ensure similar baseline behavior, an open field/eagle test (OF/E) as well as a social interaction test with conspecifics (SI/CO), as described in Milic et al. [21], was carried out with the Arc-GFP mice before the CSD. Additionally, we performed replicate experiments in parallel with WT GFP mice (Supplementary Fig. 1B–E). Mice exhibiting freezing or non-interactive behavior during the initial assessments were excluded from the study. In addition to the SI indices, we reviewed the recorded videos of the selected mice’s behavior during the SI experiment to confirm the accuracy of the SI index and to exclude any errors caused by glitches or inaccuracies in Ethovision tracking. After that, mice were split randomly into a control and a stress-exposed group (*n* = 50 control population and *n* = 90 stress-exposed populations at the start of the experiment). Sample sizes for the experiments were based on previous experiments and animal numbers used by Milic et al. [21]. With this approach, we obtained at least five biological replicates of each control, resilient, and susceptible phenotypes for optimal molecular comparisons of statistical significance.

#### Identification of individual baseline behavior for test animals

For non-biased segregation of the test subjects to control and stressed groups based on their natural interaction/freeze states, we performed the following behavior tests before CSD – (1) open field test combined with an eagle exploration (OF/E) test and (2) a social interaction test with conspecifics (SI/CO). A PC-linked overhead video camera recorded all behavioral tests, and animals were tracked with “Ethovision XT 8” (Noldus Inc., Netherlands) software. All videos were also visually assessed by an experimenter.

#### Open field and eagle exploration test (OF/E)

Experimentally naïve mice were assessed for their basal spontaneous activity in an open field grey arena (40 × 40 × 40 length, breadth, and height) [21]. First, the animal was allowed to explore the open field for 5 min in the first test. Then, the animal’s exploration after placing a toy eagle in the center of the open arena was recorded for 2.5 min.

#### Social interaction test with conspecifics (SI/CO)

Next, mice were assessed on their social target interaction as described in Milic et al. (2020), with an empty cylinder in the habituation phase and a conspecific used as a target in the test phase. See more details in the Supplementary Methods.

#### Classification of animals to control and stress groups

SI/CO scores of the test mice were listed in increasing order, and mice with consecutive scores were distributed in stressed and control groups without placing the same-parent pups into the same group. Necessary rearrangements to the list were made to ensure that each group possessed a similar mean exploration distance (derived from the OF test). Animals that froze for more than 50% of the duration when introduced to the arena during the placement of the eagle (E) or exhibited more significant than 10 s latency in interaction in the SI/CO test were excluded from further studies.

### Chronic social defeat experiments

#### Selection of aggressive CD1 mice

Prior to the beginning of the CSD procedure, CD-1 males with attack latency of less than 10 s toward male Arc-GFP mice were selected.

#### Induction of stress for the stressed group

To induce chronic social stress, we applied the same stressor as in Vennin et al. [22]. For 10 days, mice from the defeated group were subjected to three social defeat sessions (15S each) with a 30-min interval. During each session, a mouse from the stressed group was introduced into a home cage of an older, larger, and more aggressive retired male breeder of the CD-1 strain. After a cumulative physical attack for a total of 15 S, a mesh wall was introduced in the middle of the cage between the two mice, allowing sensory but not physical contact for 24 h.Twenty-four hours after the last CSD, defeated mice were placed into a new cage, remaining undisturbed for 7 days. *Handling of non-stressed controls:* mice for the non-stressed control group were handled daily for 10 days. See more details in Supplementary Methods. *Tamoxifen injection and social interaction test with CD1 mice:* animals were poked 1 day before to acclimatize them to the injection stress. Tamoxifen (TAM- 150 mg/kg, Sigma Aldrich; solvent- 1:9 of 100% ethanol: corn oil, Sigma Aldrich) was injected 5 h before SI to both stressed and non-stressed controls. SI tests were performed similarly to the procedure for the SI test with conspecifics, with the only change being the placement of a CD1 mouse in the mesh enclosure during the test phase.

Automated output from the Ethovision software was used to segregate the stressed mice into resilient and susceptible populations. In addition, the experimenter assessed all videos of candidates for the RNA-seq to increase the stringency of selection. No double blinding was performed, as initial candidate selections were based on the automated output. From within the stressed group, mice that showed a similar interaction with the CD1 mice in the SI test as the control population were assigned to the resilient population (SI index > 100), while those with a lower SI were assigned to the susceptible population (SI index < 100). Representatives from these segregated stress phenotypes of resilience and susceptibility were selected for the RNA-seq (*n* = 5–8 per condition from a pool of two mice each). In addition, similar numbers of control mice were used. To study weight changes during stress, all animals were weighed three times during the whole experimental session: (1) on the first day of CSD, (2) on the last day of CSD, and (3) on the day of SI (before TAM injection; *n* = 50 controls and *n* = 79 stressed).

### Tissue dissection and nuclei isolation

Mice were sacrificed by cervical dislocation. For nuclei isolation, tissues – PFC and vHIP were dissected according to the regions specified in Allen Brain Atlas [23]. Tissues from one to two mice were pooled in Eppendorf containing the homogenization buffer. The nuclei isolation protocol for micro-dissected tissues was performed using the protocol from Chongtham et al. [23]. The extracted nuclei were then subjected to FANS [19].

### Fluorescence activated nuclei sorting (FANS)

Flow cytometry analysis and FANS were performed using a BD FACSAria III SORP equipped with four lasers (405, 488, 561, and 640 nm) and a 70 µm nozzle. GFP expression was detected using the blue laser and a 530/30 BPfilter, whereas DAPI was detected using the violet laser and a 450/50 BP filter. Prior to sorting, 10,000 total events were recorded, and a gating strategy was applied: first, nuclei were gated according to their forward- and side-scatter properties (FSC-A/SSC-A), followed by doublet exclusion using SSC-A and SSC-W. Nuclei were then gated according to their DAPI expression. GFP expression was used as a sorting gate. Sorted nuclei were snap-frozen in a mixture of dry ice and 100% ethanol and stored at −80 °C before their respective RNA isolation extractions.

### RNA isolation and nucRNA-seq library preparation

Ten thousand GFP+ or GFP− FANS-sorted nuclei were collected in 100 μL of RLT buffer, followed by flash freezing. RNA was purified using the RNeasy Micro kit (Qiagen, Hilden, Germany) following the manufacturer’s instructions. For the nucRNA-seq library preparation, we used a ribo-depletion-based method, using the Ovation^®^ SoLo RNA-Seq System (NuGEN M01406v2, Redwood City, CA, USA). NGS library preparation was performed following NuGEN’s standard protocol (M01406v2). Libraries were prepared with a starting amount of 1.5 ng and amplified in 14 PCR cycles. The resulting cDNA was sheared using an S2-focused ultrasonicator (Covaris, Woburn, MA, USA) with the following parameters: 20% duty cycle; 0.5 intensity; 50 cycles/burst; 20 °C; 60 s. The NGS library preparation was performed with 3.16 ng of sheared cDNA with NuGEN’s Ovation Ultralow System V2 M01379 v5. Libraries were amplified in 11 PCR cycles. NGS libraries were profiled in a High Sensitivity DNA Chip on a 2100 Bioanalyzer (Agilent Technologies, Santa Clara, CA, USA) and quantified using the Qubit dsDNA HS Assay Kit, in a Qubit 2.0 Fluorometer (Life Technologies, Carlsbad, CA, USA). Samples were sequenced on NextSeq 500 Highoutput Flowcells. For PFC, reads were sequenced in a single-end manner, while for vHIP, paired-end sequencing was chosen. All RNA-seq library preparations were performed by the Genomic Core Facility from the Institute of Molecular Biology (IMB, Mainz, Germany) and sequenced in IMB or StarSEQ (Mainz, Germany).

### Nuclear RNA-seq data analysis

The data quality assessment of sequenced raw reads was performed using FASTQC (v.0.11.8). Subsequently, reads alignment was conducted to the *Mus musculus* genome (mm10) UCSC annotations using the STAR aligner (v.2.7.1a) with default parameters. Next, duplicates were eliminated from the data using the Unique Molecular Identifier introduced by NuGEN Ovation RNA Solo library. Uniquely mapped reads were retained in the output BAM file. Samtools (v1.7) [24] was employed to sort and index the mapped files. Reads count per gene was calculated using HTSeq (v0.11.1) [25]. DESeq2 Bioconductor package [26] was utilized for normalization and differential expression analysis with a default FDR. Gene ontology (GO) analysis was performed using the ToppGene database. ggplot2 R package was used for visualization. The SynGO database was used for synaptic gene analysis and sunburst plot visualization [27]. Network analysis was performed using the STRING database with a medium confidence score (0.400). Hub gene identification was done using the CytoHubba plugin from Cyctoscape software. Hub genes were identified based upon the Degree method in the network [28].

### Statistical tests for behavior experiments

All tests were performed on GraphPad Prism 9.3.0. Normality was formally tested using normality and lognormality tests in Prism. For data that were normally distributed, *t*-tests were performed. For data that were not normally distributed, the Kolmogorov–Smirnov test was performed. All tests are specified wherever necessary.

## Results

### Isolation of activated nuclei (AN) from Arc-GFP mice following the chronic social defeat (CSD) paradigm

**Fig. 1:**
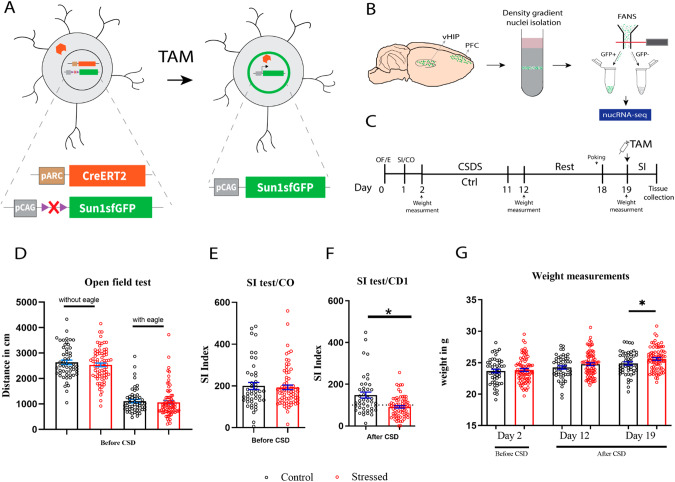
Isolation of activated neurons (AN) from Arc-TRAP/Arc-GFP mice following chronic social defeat (CSD) paradigm. **A** Schematic representation of Arc-GFP mouse line. Transgenic animals contain two transgenes including a CreER^T2^ under an activity-dependent Arc promoter (ArcCreER^T2^) and Cre-dependent fusion reporter Sun1GFP. In the presence of tamoxifen (TAM), Cre-ER^T2^ translocates to the nucleus allowing loxp site recombination and expression of the fusion nuclear membrane protein Sun1GFP. **B** Strategy for isolation of activated nuclei population. Nuclei were isolated from vHIP and PFC brain regions and sorted for GFP+ (AN) and GFP− populations using FANS. Nuclei isolation was performed from a pool of two mice per behavioral group. Sorted AN nuclei were processed for molecular assays such as nuclear RNA-seq. **C** Schematic diagram illustrating the experimental outline for CSDS (see more in Supplementary Fig. 1). Behavioral results prior to CSD experiments. Animals were segregated into control (black) and stressed (red) groups, based on their baseline behaviors of open field/eagle exploration (**D**) or social interactions with conspecifics (**E**). **F** Following CSD, the stressed group revealed a decrease in social interaction test compared to control (Kolmogorov–Smirnov test, *p* < 0.05). Animals with SI > 100 were considered as resilient and SI < 100 were considered as susceptible. **G** Stressed animals revealed significant weight gain compared to control animals (*t*-test, *p* < 0.05) on the 19th day. All error bars indicate ±SEM. Black circles represent individual non-stressed control mouse, while red circles represent individual stressed mouse.

To investigate the transcriptional alteration occurring in AN, we used pArcCreERT2(TG/WT).R26CAG-Sun1-sfGFP-Myc(M/WT) mice [16, 20] to visualize Arc-dependent neuronal activation genetically (Fig. 1A). In the presence of tamoxifen (TAM), Cre-ERT2 translocates to the nucleus, allowing loxp cassette recombination and expression of the fusion nuclear membrane protein Sun1GFP (Fig. 1A). Nuclei from the vHIP and PFC brain regions were isolated and subsequently sorted for GFP+ (i.e. AN) and GFP− populations utilizing FANS (Fig. 1B). The sorted AN were then used for nucRNA-seq for in-depth transcriptional analysis (Fig. 1B).

To assess the distinct behavioral outputs, we used a modified version of the CSD paradigm to classify susceptible and resilient animals [6] (Fig. 1C, Supplementary Fig 1A). Following the segregation of animals into control and stress-exposed groups (Fig. 1D, E, Supplementary Fig. 1B, C), CSD was performed using a stress exposure protocol similar to that described in the study by Vennin et al. [22] (see “Material and methods” and Supplementary Information, Supplementary Fig. 1B–E). Over a period of 10 days, the Arc-GFP intruder mice were subjected to 15-s attacks by the resident CD1 mouse three times per day. Following the completion of CSD on the final day, a resting period of 7 days was provided. Subsequently, an SI test was conducted using a stranger CD1 mouse as a stimulus, and SI scores were taken. Overall, we observed a significant stress effect with lower SI score in the stress-exposed cohorts (Kolmogorov–Smirnov test, *p* < 0.05, controls = 43, stressed = 73 (Fig. 1F, Supplementary Fig. 1B–D) accompanied by significant weight gain (Day 19, *t*-test, *p* < 0.05, controls = 50 and stress-exposed = 79) (Fig. 1G). Mice presenting with SI scores above 100 were designated as stress-resilient, while mice displaying SI scores below 100 were categorized as stress-susceptible. TAM was administered through injections 5 h before the SI test to capture the behavior-test-specific activated neuronal population in both stressed and non-stressed control groups. The animals were sacrificed 72 h post injections, after which the PFC and vHIP were dissected. Subsequently, a nuclei isolation procedure was conducted on the dissected brain regions for further analysis. The nuclei from two mice were pooled and GFP+ nuclei were sorted using FANS (Fig. 1B, Supplementary Fig. 2A). At this stage, we analyzed the average percentage of GFP+ nuclei within the isolated nuclei population both in the vHIP and PFC across distinct behavioral groups (Supplementary Fig. 2B). In the PFC, we observed a significantly elevated amount of GFP+ nuclei in the susceptible animals compared to control in the PFC (Supplementary Fig. 2B). A similar tendency, yet not significant, of increased neuronal activation in susceptible animals was observed in the vHIP compared to control and resilient animals (Supplementary Fig. 2B).

### GFP+ population consists of predominantly glutamatergic neurons in the vHIP and PFC

**Fig. 2:**
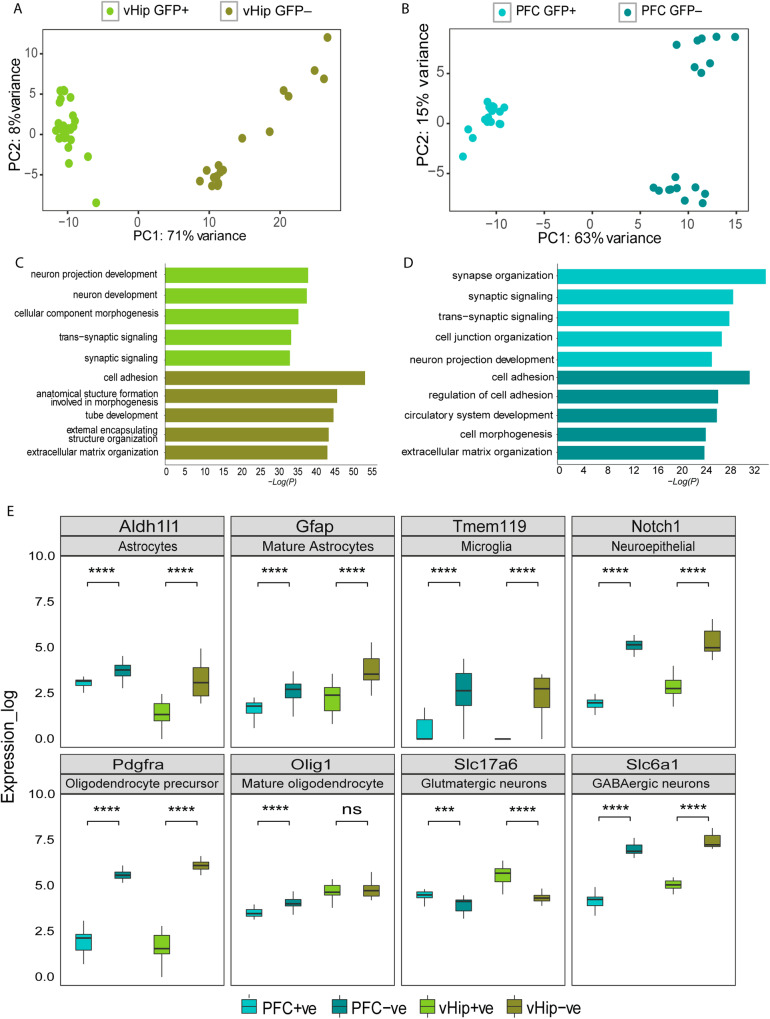
Transcriptomic characterization of Isolated GFP+ and GFP− nuclei from vHIP and PFC. Principal component analysis (PCA) of all GFP+ and GFP− samples from vHIP (**A**) and PFC (**B**). Gene ontology enrichment analysis for biological processes of GFP+ and GFP− samples in the vHIP (**C**) and PFC (**D**). **E** Gene expression of neuronal and glial markers (Astrocytes - *Aldh1I1*, Mature Astrocytes - *Gfap*, Microglia - *Tmem119*, Neuroepithelial - *Notch1*, Oligodendrocytes precursor - *Pdgfra*, Mature oligodendrocytes - *Olig1*, Glutamatergic neurons - *Slc17a6*, GABAergic neurons - *Slc6a1*) across GFP+ and GFP− samples in the vHIP and PFC (***p < 0.001, ****p < 0.0001, ns non-significant).

Next, we opted to characterize the transcriptional states of TAM-activated nuclei from resilient, susceptible, and non-stressed control mice in the vHIP and PFC. Using nucRNA-seq, we compared the GFP+ and GFP− nuclei transcriptional states to determine if the samples originated from distinct cellular populations. Principal component analysis (PCA) revealed two distinct clusters of the GFP+ and GFP− populations (Fig. 2A, B). We performed differential expression analysis followed by GO analysis to identify mechanisms enriched in the GFP+ and GFP− populations (Fig. 2C, D, Supplementary Tables 1, 2). Among the top five most enriched GO terms for biological processes, we identified terms such as “cell adhesion” and “extracellular matrix organization,” which were enriched in both vHIP and PFC of the GFP− population (Fig. 2C, D, Supplementary Table 2). In contrast, the top five GO terms of the GFP+ populations in vHIP and PFC revealed terms such as “neuron projection development” and “cellular component morphogenesis” enriched in vHIP, while “synapse organization” and “cell junction organization” enriched in the PFC (Fig. 2C, D, Supplementary Table 2).

Next, we sought to determine the expression of defined brain cell types using specific markers of neural/glial cells within the GFP+ and GFP− populations (Fig. 2E). Of note, only the expression of the glutamatergic neuronal marker *Slc17a6* was significantly elevated in the GFP+ population as compared to GFP−, both in the vHIP and PFC (Fig. 2E). Expression of glial and epithelial markers as well as markers for inhibitory neurons was significantly elevated in the GFP− populations (Fig. 2E). Expression of *Olig1*, however, revealed no significant difference between GFP+ and GFP− populations in vHIP, suggesting that the GFP+ nuclei, besides glutamatergic neurons, also contain mature oligodendrocytes. To examine further the possibility of apprehending oligodendrocytes within the GFP+ population, we measured the expression of additional oligodendrocyte markers such as *Olig2, Sox10*, and *Mog* (Supplementary Fig. 2C). Interestingly, the expression of *Sox10* and *Mog* was significantly elevated in the GFP+ compared to the GFP− nuclear populations suggesting further that the GFP+ active nuclei indeed contained oligodendrocytes (Supplementary Fig. 2C). To assess if the oligodendrocytes within the GFP+ populations are rare cellular species expressing *Arc*, we checked the transcriptional co-expression of selected markers alongside *Arc* on a single cell level using the available “Allen Brain Map” database [29]. Indeed, co-expression of *Arc* within a small subset of oligodendrocytes was seen (Supplementary Fig. 2D, E). A single-cell RNA-seq study with oligodendrocytes isolated from several neurodegenerative disease models also detected a cluster of mature oligodendrocytes expressing *Arc* [30]. Therefore, it is likely that we have captured rare *Arc*-expressing non-glutamatergic populations, including oligodendrocytes, within the AN. Nevertheless, our results suggest that the captured GFP+ population consists primarily of excitatory neurons, whereas the GFP− consists of varying cellular populations present in the mammalian brain.

### Differential expression in AN of resilient vs. susceptible animals in the vHIP

**Fig. 3:**
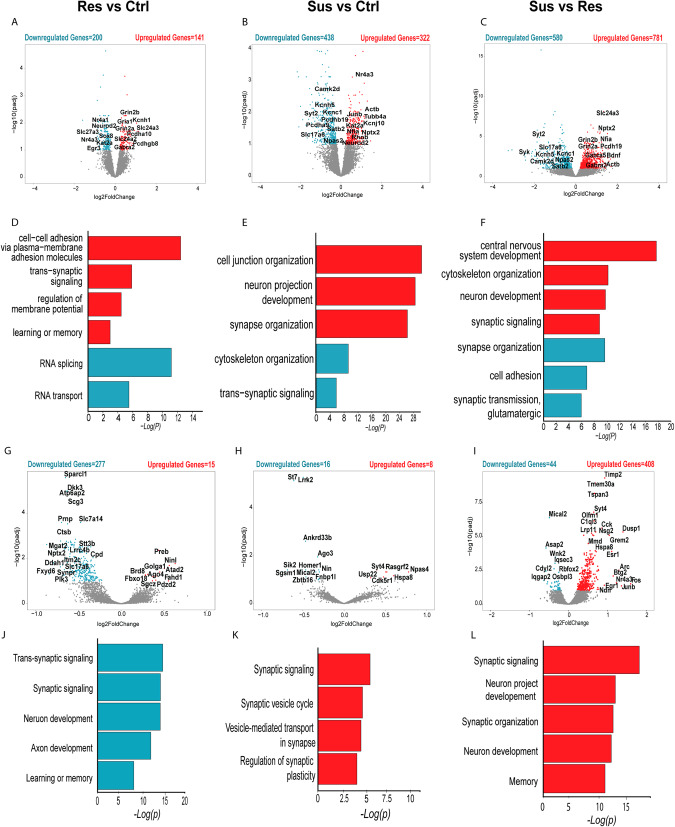
Characterization of DEGs in the vHIP and PFC between the distinct behavioral groups. Volcano plots representing the upregulated (red) and downregulated (blue) DEGs in Res vs. Ctrl (**A**), Sus vs. Ctrl (**B**), and Sus vs. Res (**C**) in vHIP. A total of 6–8 samples per condition, FDR < 0.1. GO term analysis of R vs. C (**D**), S vs. C (**E**) and S vs. R (**F**). Volcano plots representing the upregulated (red) and downregulated (blue) DEGs in Res vs. Ctrl (**G**), Sus vs. Ctrl (**H**) and Sus vs. Res (**I**) in PFC. GO term analysis of R vs. C (**J**), S vs. C (**K**) and S vs. R (**L**). A total of 4–5 samples per condition, FDR < 0.1.

To characterize further the different transcriptional programs of the AN in the different behavioral groups, we first profiled the differentially expressed genes (DEGs) in the AN of resilient, susceptible, and control animals in the vHIP and PFC. The comparison profiles included the following conditions: resilient vs. control [R vs. C], susceptible vs. control [S vs. C], and susceptible vs. resilient [S vs. R] (Supplementary Table 3, Supplementary Fig. 3A–D). In the vHIP, R vs. C comprised 200 downregulated and 141 upregulated genes (Fig. 3A), and S vs. C comprised 438 downregulated and 322 upregulated genes (Fig. 3B). S vs. R exhibited the largest number of DEGs with 580 downregulated and 781 upregulated genes (Fig. 3C). Using GO analysis, we identified condition-enriched biological processes associated with each of the behavioral groups. Resilience-associated upregulated DEGs were enriched for “cell adhesion” and “synaptic transmission, glutamatergic” and “synaptic signaling” (Fig. 3D, Supplementary Table 4). Noticeably, we observed several DEGs downregulated in resilient animals when compared to control associated with “RNA splicing” and “mRNA processing” (Fig. 3D, Supplementary Table 4). In contrast, the susceptible group displayed upregulated DEGs compared to controls, which were enriched for processes related to “synapse organization” and “cell junction organization,” while the downregulated DEGs were enriched for “cytoskeleton organization” and “trans-synaptic signaling” (Fig. 3E, F). When comparing DEGs upregulated in susceptible vs. resilient animals, we found enrichment in processes related to “cytoskeleton organization” and “synaptic signaling,” while the downregulated DEGs were enriched for “cell adhesion” and “synapse organization” (Fig. 3C, F, Supplementary Table 4). These findings demonstrate an intricate molecular interplay with cell adhesion genes, showing an overall upregulation in resilient animals compared to other groups, while cytoskeleton organization genes are upregulated in susceptible animals compared to resilient but downregulated compared to control animals. On the other hand, synapse organization genes are upregulated in susceptible animals compared to control animals but downregulated compared to resilient animals. Proper synapse organization is crucial for effective neural communication, while cytoskeleton morphology contributes to neuronal growth and the transport of essential components, ultimately impacting overall neuronal activity and function. These differential expression patterns in cell adhesion, cytoskeleton organization, and synapse organization genes in the vHIP may collectively contribute to the distinct behavioral outcomes observed in resilient and susceptible animals.

### In the PFC, susceptible AN reveal a widespread upregulation of genes associated with synaptic signaling and activity

In the PFC, the R vs. C comparison group comprised 277 downregulated and 15 upregulated genes, S vs. C 16 downregulated and 8 upregulated, and S vs. R consisted of 44 downregulated and 408 upregulated genes (Fig. 3G–I, Supplementary Fig. 3E–H, Supplementary Table 3). Due to the reduced number of DEGs, GO enrichment analysis was limited to the identified DEGs between the distinct conditions. Nevertheless, similarly to vHIP, synapse-associated genes were enriched in all the compared conditions (Supplementary Table 3). Genes downregulated in resilient animals compared to control were enriched for “synaptic signaling” and “neuron development” (Fig. 3J). Susceptibility-specific upregulated genes were enriched for “synaptic signaling”, “synaptic vesicle cycle”, and “regulation of synaptic plasticity” (Fig. 3K, L). These results suggest that in the PFC, the general upregulation of genes in the susceptible group might signify amplified synaptic activity and signaling, potentially indicating increased cognitive processing. In contrast, the downregulated DEGs associated with synaptic signaling and neuron development in the resilient group could suggest a more restrained synaptic function and cellular maturation, potentially reflecting a more stable and balanced neural state under chronic stress conditions.

### Gene families specifically upregulated in AN of the vHIP of resilient and susceptible animals

Next, we investigated distinct gene families showing differential expression in the AN of distinct behavioral groups, aiming to identify shared genes that exhibit coordinated changes across all conditions (Supplementary Fig. 4A–C). Additionally, we clustered the gene families according to their occurrence and behavioral classification and focused our analyses on the DEGs that were upregulated in susceptible or resilient animals (Supplementary Figs. 5–7). In the vHIP, the R vs. C upregulated DEGs revealed gene families enriched mainly for “clustered protocadherins (cPcdhs)”, a class of cell adhesion molecules known to play a role in neuronal survival and dendritic self-avoidance [31] (Supplementary Figs. 4A, 5E, 6A). On the other hand, in S vs. C, we observed that “non-clustered protocadherins (ncPcdhs)” were upregulated. Although structural molecules similar to “cPcdhs”, these are known to be involved in neural circuit formation and maintenance [32] (Supplementary Fig. 6B). The upregulation of distinct protocadherin families in the vHIP of susceptible and resilient animals suggests two separate pathways of cell adhesion molecules to perform distinct functional roles. Additional notable gene families displaying distinct expression patterns comprise “glutamate ionotropic receptor kainate type subunits,” which were upregulated in the resilient group compared to both the control and susceptible groups (Supplementary Figs. 4A, C, 5E). Among the R vs. C downregulated gene families, we found a large number of genes with an RNA binding motif, which is in line with the observed enrichment of RNA processing mechanisms in the GO terms (Supplementary Figs. 4A, 6A). Among the gene families that were upregulated in S vs. R, we identified several gene families belonging to the “Actins”, “Tubulins”, and “Rho family GTPases” known to be involved in cytoskeleton remodeling mechanisms [33] (Supplementary Figs. 5B, 6C).

**Fig. 4:**
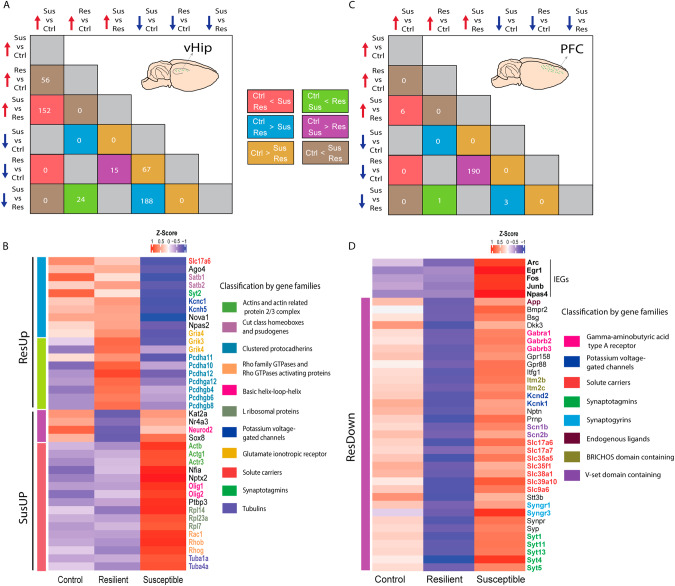
Identification of most significant DEGs in the vHIP and PFC and classification according gene families between the distinct behavioral groups. **A** Overlapping DEGs across the distinct behavioral conditions classified as upregulated in susceptible compared to resilient (red and purple) and resilient compared to susceptible (green and blue). **B** Heatmap of nuclear RNA-Seq expression *z*-scores computed for selected differentially expressed genes between the behavioral groups in the vHIP. **C** Overlapping DEGs across the distinct behavioral conditions classified as upregulated in susceptible compared to resilient (red and purple) and resilient compared to susceptible (green and blue). **D** Heatmap of nuclear RNA-Seq expression *z*-scores computed for selected differentially expressed genes between the behavioral groups in the PFC.

Throughout our data, we observed alterations in multiple DEGs across several comparison groups, prompting us to perform an overlap analysis to identify shared gene targets that exhibit changes in expression corresponding to the behavioral conditions. This approach allowed us to emphasize the most significant gene targets that were consistently altered in more than one comparison group and focus mainly on the differences between resilient and susceptible groups. In vHIP, we identified a total of 167 upregulated genes in susceptible and of 216 upregulated genes in resilient animals. These groups were termed susceptible-up (SusUp) and resilient-up (ResUp) (Fig. 4A, Supplementary Fig. 6D, and Supplementary Table 5). Among the most significant DEGs are previously described gene families such as “Actins,” “Tubulins,” “Rho family GTPases” appearing in the SusUp group and “cPcdhs” and “Glutamate ionotropic receptor kainite type subunits” appearing in ResUp (Fig. 4B). These findings highlight the predominant association of upregulated cell adhesion, particularly the “cPcdhs” gene family, with resilient behavior, while cytoskeleton remodeling gene families are specifically upregulated in the vHIP of susceptible animals.

### Specifically upregulated gene families suggest increased synaptic activity in PFC AN of susceptible animals

Using a comparable approach to the vHIP analysis, we explored the differential expression of specific gene families in the AN of the PFC across distinct behavioral comparisons. Interestingly, we observed the enrichment of several gene families that were downregulated in the R vs. C yet upregulated in the S vs. C and/or S vs. R conditions. These gene families, including “Gamma-aminobutyric acid type A receptor subunits”, “solute carriers,” “ATPase Na+/K+ transporting subunits,” and “synaptotagmins” (Fig. 4D, Supplementary Fig. 7). Synaptotagmins are known to play a role in calcium-dependent presynaptic neurotransmitter release. The systematic downregulation of this gene family in the resilient animals suggests a decrease in inner calcium levels concentration and, in line with the observation of an amplified neuronal activity in the susceptible animals, potentially amplified neuronal activity [34, 35]. These alterations are associated with synaptic transmission, neurotransmission, and ion transport (Supplementary Fig. 4D–F). The upregulation of these gene families in the susceptible condition suggests an increase in neuronal activity and altered synaptic signaling.

Next, we performed an overlap analysis of DEGs identified in distinct behavioral conditions within the PFC to identify shared gene targets displaying changes in expression associated with the behavioral conditions. Here, we identified a total of 196 resilient-downregulated (ResDown) and 4 ResUp genes (Fig. 4C, Supplementary Table 5). We clustered the gene families according to their occurrence and behavioral classification and identified three main groups that were associated with more than one behavioral comparison (Supplementary Fig. 7). Most notably, 21 gene families were downregulated in R vs. C and upregulated in S vs. R, all classified within the ResDown group in the PFC (Supplementary Fig. 7). Interestingly, we observed an upregulation of various IEGs including *Arc, Fos, Junb, Npas4,* and *Egr1* (Fig. 4D). The observed upregulation of IEGs in susceptible AN suggests a hyperactivated neuronal response associated with the susceptible condition. These findings collectively indicate that the AN in the PFC of susceptible animals undergoes activity-dependent neuronal changes, while the AN in resilient animals remains relatively unaffected or less activated.

### Protein–protein interaction analysis identifies distinct hub genes linked to susceptibility and resilience

Following our observations of enriched mechanisms and specific gene families associated with cytoskeleton organization and synaptic regulation in the distinct behavioral groups, we opted to characterize the protein–protein interaction (PPI) network of the identified SusUp or ResUp groups. To understand the possible PPI of these DEGs and to identify hubs, we obtained networks from the STRING database. Interestingly, in the vHIP SusUp gene network, we found that *Rac1* and *Actb* act as hub genes based on the degree of connectivity to other genes (Supplementary Fig. 8A). In contrast, in ResUp, we identified *Gria4* and *Syk* as hub genes (Supplementary Fig. 8B). *Rac1* and *Actb*, both implicated in cytoskeleton organization, may serve as critical hub genes influencing susceptibility to stress. On the other hand, *Gria4*, alongside *Grik3* and *Grik4*, encode subunits of ionotropic glutamate receptors, which are essential constituents of glutamate excitatory neurotransmission. Their upregulation in AN of resilient animals may enhance excitatory neurotransmission mediated by AMPA and kainate receptors, which leads to modifications in synaptic transmission and synaptic plasticity. *Syk* has been established as a crucial component in adaptive immune receptor signaling [36]. However, emerging evidence suggests that *Syk* also plays a role in other diverse biological functions, such as cellular adhesion and innate immune recognition. In the ResDown group in the PFC, we identified *Prnp* as a hub gene, as it exhibited a high degree of connectivity to other genes within the network (Supplementary Fig. 8C). *Prnp* has previously been implicated with adaptive stress responses to acute stress [37] and its downregulation in AN of resilient animals may confer a more significant role in the establishment of the resilient phenotype than previously recognized. Taken all together, our results highlight the role of specific hub genes in regulating cytoskeleton organization and synaptic neurotransmission as key mechanisms underlying the establishment of either susceptible or resilient behavior.

### Synapse-specific alteration in distinct synaptic functions between susceptible and resilient AN

**Fig. 5:**
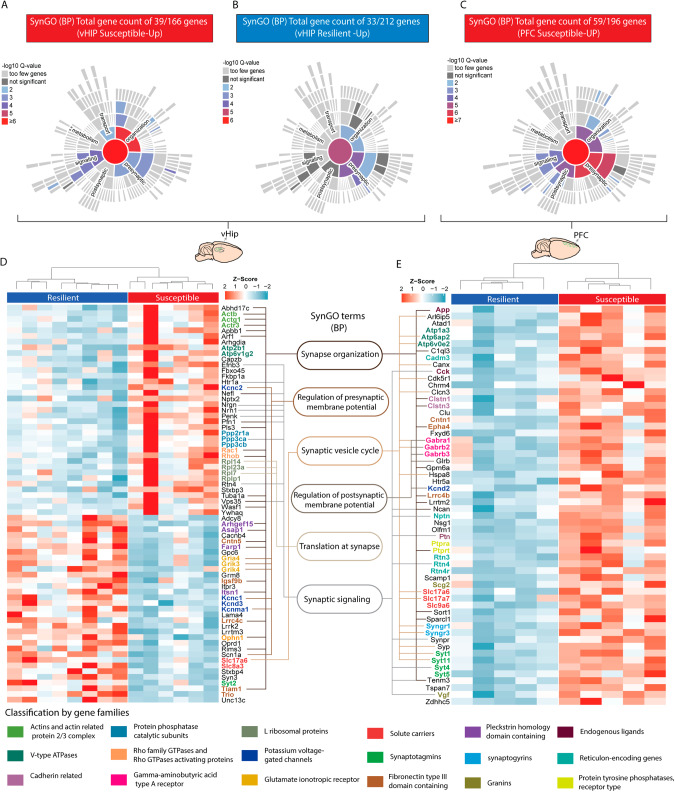
Identification of synaptic-specific DEGs associated with resilient and susceptible AN, both in the vHIP and PFC. Sunburst plots of gene enrichment for Susceptible-up (**A**) and Resilient-up (**B**) DEGs in vHIP and Susceptible-up (**C**) in PFC as denoted by SynGO [27]. Significantly enriched for biological processes are indicated by color code at 1% FDR (at least three matching input genes). Database entries for cellular components and for biological processes were considered as indicated in Supplementary Table 6. Heatmap of 39 susceptible-up and 33 Resilient-up DEGs in vHIP (**D**) and 59 susceptible-up DEGs in PFC (**E**) classified according to their gene family. Gene expression in is represented by expression *z*-scores computed for selected DEGs. Six enriched Synaptic Gene Ontology (SynGO) terms are illustrated in the middle with the corresponding genes connected by a line.

Following our observations of enriched mechanisms and specific gene families associated with cytoskeleton organization and synaptic regulation in the distinct behavioral groups, we opted to characterize further the behavior-specific (susceptible or resilient) transcriptional programs related to synaptic functions. For such analysis, we focused on the most significant gene targets altered in more than one comparison group in the vHIP and PFC (Fig. 4B, D). With the classified group-specific DEGs, we performed synapse-specific analysis using SynGO, an online knowledgebase analysis platform focusing on functional annotation of synapse-specific GO terms [27]. In the vHIP, we identified 39 synapse-associated genes from the 167 genes upregulated in susceptible animals (Fig. 5A, Supplementary Table 6) and 33 out of the 261 genes upregulated in resilient animals to be annotated to a synaptic function (Fig. 5B, Supplementary Table 6). In the PFC, however, 60 out of the 196 genes upregulated in susceptible animals were synapse-associated (Fig. 5C, Supplementary Table 6). Functional annotation of the upregulated synaptic genes in the susceptible group in the vHIP to biological processes revealed enrichment for “synapse organization”, “synaptic signaling,” and “regulation of synaptic vesicle endocytosis” (Fig. 5A, Supplementary Fig. 9, Supplementary Table 6). On the contrary, synaptic genes upregulated in the resilient group were enriched for “process in presynapse” and “regulation of presynaptic membrane potential” (Fig. 5B, Supplementary Fig. 9, Supplementary Table 6). In the PFC, however, functional annotation of synapse-associated genes upregulated in the susceptible group were enriched for “presynapse” and “integral component of synaptic vesicle membrane” (Fig. 5C, Supplementary Fig. 10, Supplementary Table 6). Among the synapse-associated DEGs, we identified redundant gene families, consistent with our prior findings linked to susceptible and resilient behavioral phenotypes (Fig. 5D, E). In the vHIP, susceptible animals exhibit transcriptional profiles primarily characterized by neuronal cytoskeletal remodeling processes, whereas resilient animals display transcriptional signatures related to the regulation of membrane potential and synaptic activity (Fig. 5D). Conversely, in the PFC of susceptible animals, there is a notable upregulation of genes associated with synaptic function, indicative of an overall increased synaptic activity compared to resilient and control animals (Fig. 5E). Collectively, our findings underscore distinct synaptic mechanisms underlying susceptible and resilient behavior. Susceptible animals demonstrate elevated levels of neuronal and structural alterations in the vHIP, along with widespread transcriptional changes linked to neuronal activation in the PFC. In contrast, resilient animals exhibit transcriptional profiles associated with synaptic plasticity and a reduced stress-induced neuronal activation response.

## Discussion

Chronic stress has become a predominant factor associated with a variety of disorders [38]. Therefore, it is essential to explore the molecular mechanisms related to the behavioral alterations to chronic stress. To do so, we used the CSD model, where we observed a successful stress effect of eliciting learned avoidance behavior [21, 39, 40]. It is important to note that in most studies using this model, the SI test was performed 24 h after the last CSD. However, stress effects have been shown to persist for at least a month [6, 16, 39, 41] (see more details in Supplementary Information). Based on the disease trajectory of PTSD, we were interested in the chronic changes caused by CSD and have therefore performed SI testing 7 days after stress exposure in this study. Induction of Arc expression through tamoxifen injection immediately before the SI task (on day 8) made monitoring such chronic effects of stress on SI behavior possible. Our study provides a robust experimental design to study long-term retention of stress memories.

We observed a gradual increase in body weight in the stressed populations, determined as significantly different from the control group. The gradual increase in body weight could be due to slow accumulating changes in metabolism following stressful events and is in line with other observations of weight gain after stress [42]. However, some studies using similar models have reported contradictory findings on weight loss [43, 44] and gain [45–47] following exposure to social defeat stressors. It would be important to understand the impact of the stress caused by particular behavioral paradigms on the alteration of metabolic pathways, which ultimately affects body weight.

Multiple studies have looked at transcriptomes after social defeat stress. However, they mostly focused on bulk tissue sequencing, which can dilute important molecular signatures in cells activated by SI challenges. Therefore, in our study, we utilized animals with an activity-dependent Sun1GFP reporter, driven by the *Arc* promoter, to monitor and sort activated neuronal populations. Through this approach, we conducted a comparative analysis of transcriptional signatures between AN and non-AN derived from resilient and susceptible animals, providing insights into the molecular alterations underlying individual stress responses. Recent studies using similar strategies helped to identify the chromatin structure dynamics underlying neuronal activation during epileptic seizures [17], during memory formation [18], and an instability stress paradigm using female mice [48]. Arguably and in accordance with the experience-dependent neuronal activation theory [15, 49, 50], the collected AN are associated with the recall of the stress exposure (SI with CD1). Therefore, the molecular associations identified in this study provide a novel view of stress-recall activated cellular and molecular response mechanisms. Studying the specific role of the stimulated neuronal populations is arguably a fitting strategy to comprehend further the influence of the investigated cellular population on the overall behavioral phenotypes.

Here, we examined the active nuclei populations isolated from vHIP and PFC, two highly investigated brain regions shown to be associated with the manifestation of mood-related disorders [33, 51–57]. Our results indicate that the captured GFP+ population predominantly consists of excitatory neurons, while the GFP− population comprises various cellular populations found in the mammalian brain. However, we propose a plausible explanation for the presence of rare Arc-expressing non-glutamatergic cells within the GFP+ population, potentially originating from mature oligodendrocytes. Notably, we observed elevated expression levels of oligodendrocyte markers in susceptible AN of the vHIP relative to resilient animals. This finding suggests a potential involvement of Arc-expressing oligodendrocytes in stress susceptibility. Previous studies have indicated that susceptible animals undergo active adaptation processes in response to stress exposure. For instance, Vennin et al. [22] employed a modified SI test followed by single-cell RNA-seq, revealing a subgroup of mice within the traditionally classified susceptible group (termed intermediate), which exhibited an active and dynamic non-neuronal molecular response associated with brain restoration and homeostasis, potentially contributing to adaptation and stress resilience.

**Fig. 6:**
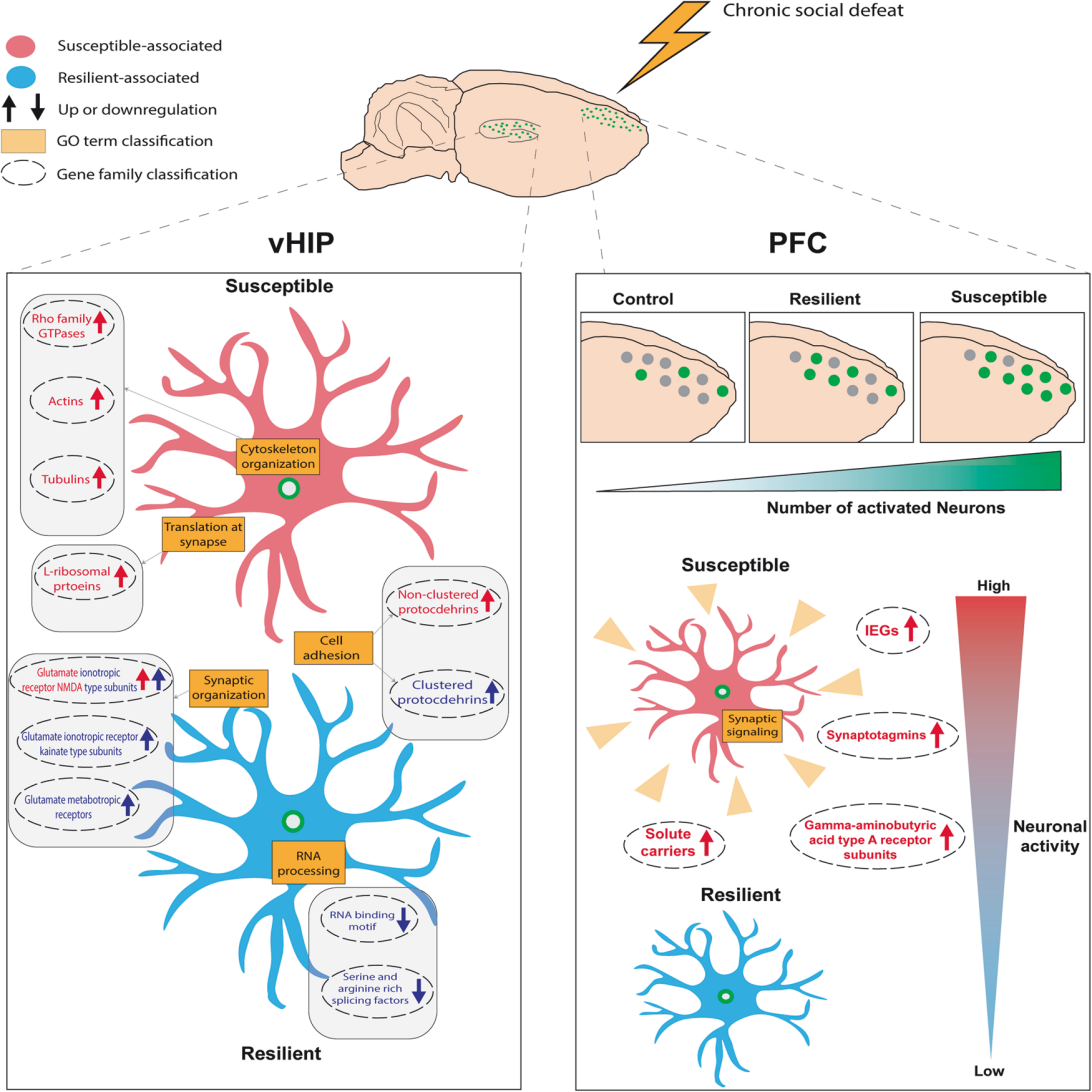
Summary of the transcriptional changes observed in activated nuclei associated with susceptible and resilient within vHIP and PFC. In the ventral hippocampus (vHIP), transcriptional changes associated with susceptibility are characterized by alterations in synaptic cytoskeleton organization and non-clustered protocadherins. On the other hand, transcriptional changes associated with resilience involve an increased expression of various glutamate ionotropic and metabotropic receptors, as well as clustered protocadherins, while the expression of RNA binding and serine/arginine splicing factors is decreased. In the prefrontal cortex (PFC), susceptible animals display an overall increased number of activated nuclei as well as an overall increase in expression, primarily observed in synaptic signaling and immediate early genes.

In our extensive transcriptional analyses of AN (GFP+), we observed substantial alterations in gene targets within the vHIP and PFC between susceptible and resilient animals (Fig. 6). In the vHIP, AN of susceptible animals exhibited increased expression of genes related to cytoskeleton organization, such as actins, tubulins, and Rho GTPase family members known to modulate cytoskeleton reorganization [33, 58, 59]. Many of these genes have been implicated in synaptic function and suggest that excitatory neurons in the vHIP and the PFC in susceptible animals undergo cytoskeletal reorganization. Previously it had been suggested that the actin cytoskeleton is a key regulator of synaptic receptor activation during learning and memory in the hippocampus and amygdala [60]. We here identified an intricate interplay between “cytoskeleton organization” genes, which were upregulated in susceptible animals compared to resilient, and “synapse organization” genes which were downregulated in susceptible animals compared to control animals but upregulated compared to resilient animals in a CSD model that is related to classical fear condition models and thus fear memory [40]. Furthermore, we identified unique expression patterns for various genes associated with glutamatergic signaling in the vHIP, including several ionotropic glutamate receptors. These genes directly participate in the release and uptake of neurotransmitter vesicles, underscoring their relevance to synaptic organization and transmission mechanisms [61, 62]. For instance, “Glutamate ionotropic receptor NMDA type subunits” (*Grin2a, Grin2b*) were upregulated both in susceptible and resilience compared to control whereas “Glutamate ionotropic receptor kainate type subunits” (*Grik3, Grik4*) and “Glutamate metabotropic receptor” (Grm1, Grm3) were upregulated in resilient animals compared to susceptible and control or only control, respectively. The interacted interconnection of various subunits within the glutamate receptors suggests a complex network that undergoes distinct alterations within the susceptible or resilient activated neurons.

In both resilient and susceptible animals, we observed cell adhesion molecules upregulated in vHIP. However, cPcdhs were upregulated in resilient animals, while susceptible animals had cell adhesion molecules of the ncPcdhs gene family, including *Pcdh8, Pcdh17, Pcdh19*, and *Pcdh20*, upregulated in their AN. Protocadherins are cell adhesion molecules expressed widely in the central nervous system and are involved in several neuron-related functions [32]. Several studies have linked altered expression of both, cPcdhs and ncPcdhs with mental disorders such as bipolar disorder [63], schizophrenia [64, 65], and major depressive disorder [66–68]. While cPcdhs have been associated with processes assigned to the functionality of single neurons, including neuronal survival, dendritic self-avoidance, neural identity diversification, and synaptogenesis [31, 66], ncPcdhs seem to affect the entire network and regulate neural circuit formation and maintenance [32]. Our data suggest that in resilient animals the stress activates transcriptional programs that stimulate neuron diversity and extend the neuronal communication system potential mediated via increased expression of cPcdhs, focusing on strengthening synaptic connection. On the other hand, the transcriptional programs of susceptible animals involve extensive neural remodeling via cytoskeletal organization mechanisms potentially to adapt to stress exposure. These differential expression patterns in cell adhesion, cytoskeleton organization, and synapse organization genes in the vHIP may collectively contribute to the distinct behavioral outcomes observed in resilient and susceptible animals. Further studies are required to study the role of network vs. single neuron adaptations in resilient and susceptible animals, particularly in activated neuronal populations following stress exposure. Techniques such as spine imaging or electrophysiology experiments could be sufficient to answer such questions and shed light on the mechanistic aspects of protocadherins within the context of stress exposure.

The transcriptional profiles observed in the PFC revealed a general predominant gene upregulation in susceptible AN compared to control and resilient animals. Additionally, susceptible mice exhibited a greater degree of neuronal activation in the PFC compared to the vHIP, with a significant increase observed in the active neural population of the PFC in susceptible animals compared to control (Fig. 6, Supplementary Fig. 2B). For instance, various genes from the “synaptotagmins” gene family including *Syt1*, *Syt4*, *Syt5*, *Syt11*, and *Syt13* were upregulated in AN of susceptible compared to resilient animals in the PFC (Supplementary Fig. 5). Synaptotagmins are known to play a role in calcium-dependent presynaptic neurotransmitter release and the systematic increase of this gene family might suggest an increase in inner calcium levels concentration and potentially amplified neuronal activity [34, 35]. Notably, the upregulation of IEGs, including *Arc*, *Fos*, *Junb*, *Npas4,* and *Egr1* supports the notion of increased neuronal activity and synaptic plasticity in the PFC of susceptible mice [13, 15, 67]. Previous studies examining the bulk expression of IEGs such as *Arc,* *Fos*, and *Egr1* in the PFC and vHIP revealed decreased activity in vHIP associated with resilient behavior and decreased activity in the PFC associated with susceptible behavior [7, 8]. However, recent single-cell data found the opposite, particularly when considering specific neuronal populations [68]. Furthermore, in line with our data, a study examining layer 2/3 excitatory neurons in the medial PFC showed increased synaptic potentiation within the activated neurons in susceptible mice exposed to a learned helplessness paradigm. In contrast, weakened synaptic potentiation was associated with resilient mice [69]. Such studies emphasize the importance of studying cell-type specific populations within heterogeneous brain regions, as compared to generalized bulk studies.

Our study has provided valuable insights into the impact of stress on activated neurons. However, it is crucial to acknowledge certain limitations. We presented comprehensive transcriptional profiles delineating differences among distinct behavioral groups. Nevertheless, future studies should investigate the mechanistic nature of our findings in more detail. For instance, using genetically modified viruses in activated neurons could enable the manipulation of gene expression, allowing for a closer examination of potential changes in behavioral functions. Second, we recognize that our findings primarily stem from experiments conducted on male mice, given that CSD stress is predominantly applied to males. As significant sex differences have been observed in the stress response and stress-mediated effects on behavior, our approach leaves out important findings concerning female animals [70–72]. Therefore, exploring the impact of stress on neuronal activation within behavioral paradigms that include both sexes is crucial. For example, a paradigm of chronic variable stress (CVS) can be applied to both males and females, offering a more comprehensive understanding of stress impacts [73, 74]. Lastly, despite using the CSD as a robust mouse model of stress-resilience, recent studies have challenged the previous notions of resilience and susceptibility to CSD, highlighting a more intricate behavioral and neurobiological response to stress [49, 75]. Therefore, it is crucial to investigate diverse behavioral paradigms with clearly defined behavioral states to understand how stress can influence and modify behavior. The integration of these modified behavioral tests and the subclassification of animals, along with the strategy of sorting and analyzing AN, holds promise for providing a comprehensive understanding of the complex stress-related responses and the dynamics within specific activated cell populations.

## Supplementary information

Supplementary information

Data Set 1

Data Set 2

Data Set 3

Data Set 4

Data Set 5

Data Set 6

## Data Availability

All sequencing data generated or analyzed during this study are available on NCBI GEO under accession no. GSE240573. All other data are included in this article (and its supplemental data files).
