# Supplementary material for: Characterization of transcriptional profiles associated with stress-induced neuronal activation in Arc-GFP mice
Source: Mol Psychiatry. 2024 Apr 22;29(10):3010–23. doi: 10.1038/s41380-024-02555-z (PMC11449785; doi:10.1038/s41380-024-02555-z)
Supplement: Supplementary file 1 — Supplementary information [file 41380_2024_2555_MOESM1_ESM.docx]

**Supplementary information**

**Supplementary Experimental Procedures:**

Genotyping

Genotyping was performed using the following primers: Arc creERT2: Common forward primer – 5’-CAG CAT AAA TAG CCG CTG GT-3’; Wild type reverse primer – 5’-CCG TCC AAG TTG TTC TCC AG-3’; Transgenic reverse primer – 5’-CGA CCG GTA ATG CAG GC-3’. Fragments for WT/WT allele and TG/WT allele are detected at 300 bp and 300 bp + 500 bp, respectively. Sun1GFP: Common forward primer – 5’GCA CTT GCT CTC CCA AAG TC 3’; Wild type reverse primer – 5’CAT AGT CTA ACT CGC GAC ACT G3’; Mutant reverse primer – 5’GTT ATG TAA CGC GGA ACT CC 3’. Fragments for WT/WT allele, M/WT (GFP/WT) allele and M/M (GFP/GFP) allele are detected at 557 bp, 300 bp + 557 bp and 300 bp respectively.

Social Interaction Test

In the habituation phase, test mice were introduced at the corner opposite the cylindrical empty mesh enclosure. The test mouse was allowed to explore the arena and the mesh enclosure for a total of 150 S. Ethovision XT 15 (Noldus) system was used for video-tracking to measure the total time of presence of mouse nose in the interaction zone, defined as an 8 cm interaction zone. Subsequently, the test mouse was temporarily placed in a bedding environment, while a conspecific/CD1 mouse was introduced into the mesh enclosure in the arena. Shortly afterwards, during the test phase, the test mouse was placed in the arena and the interactions were recorded (for 150 S), similar to the habituation phase. Social interaction (SI) index was then calculated as the ratio of time spent exploring the interaction zone during the test phase/time spent exploring the interaction zone during the habituation phase (× 100).

NB: Mice that were severely wounded during the stress paradigm or mice that exhibited abnormal behavior during the interim of the experiments were removed before the SI test. Apart from this, animals that exhibited a >10 S latency for interaction during the SI test were excluded from the final number of animals, reducing the numbers for the SI test to n = 43 controls and n = 73 stressed for Arc-GFP mice.

Handling of control mice

Control mice were also housed in similar conditions as stressed mice with the only difference being a conspecific mouse on the other side of the mesh. During the daily handling, control mice were allowed to explore the in-house conspecific for about 15 S, repeated for a total of 3 times. All cages were maintained in environmentally controlled cabinets (Uniprotect NG, Zoonlab GmbH), which were located in different rooms for stressed and non-stressed groups. Next day, after the last defeat session all animals were separated and housed individually before the SI.

Experimental design of the chronic social defeat (CSD)

In our study we employed the CSD paradigm, where the experimental mice were subjected to daily three rounds of defeats for 15 S each, repeated over a span of 10 days. Following 7 days recuperation period the SI test was performed where we observed successful stress-effect of eliciting learned avoidance behavior [Milic et al., 2020, Ayash et al., 2020, Wendelmuth et al., 2020]. In our experimental design we included 7 days recuperation time to: 1. allow a recuperation period to heal the physical wounds that could impact behavior and 2. minimize the interference of artificial estrogen (TAM) during or shortly after the stress induction 3. follow the mechanisms pertaining towards the long-lasting effects of stress (recall). It is important to note, most of the studies using CSD, the SI test is performed 24 hours after the last CSD. However, stress effects have been shown to persist for at least a month [Krishnan et al., 2007, Bagot et al., 2016, Harris et al., 2018, Wendelmuth et al., 2020], which was also noted in our pilot experiments (data not shown). Considering the enduring effects of stress, the CSD and SI tests may offer valuable avenues for investigating post-traumatic stress disorders, particularly those arising from robust memory encoding of traumatic events, such as social defeat in our study. Apart from the behavioral detection of the persistence of stress effects, we also observed a gradual increase in body weight in the stressed populations, determined as significantly different from the control group. The gradual increase in body weight could be due to the slow accumulating changes in metabolism following stressful events.

Regarding the TAM injections, they were deliberately timed to coincide with stress recall (7 days later) rather than the stress induction itself. This decision stems from concerns about potential interactions between TAM and immediate stress effects, as indicated in previous research (Li et al., 2020). We were cautious about the possibility that TAM injection immediately after stress exposure could alter stress readouts and influence susceptibility-resilient effects. Opting to administer TAM during stress recall ensures that the resulting stress effects are stabilized. Our unique experimental design, focusing on molecular mechanisms 7 days post-CSD, provides a rare opportunity to explore the sustained changes following the social defeat.

**Supplementary figures:**

**
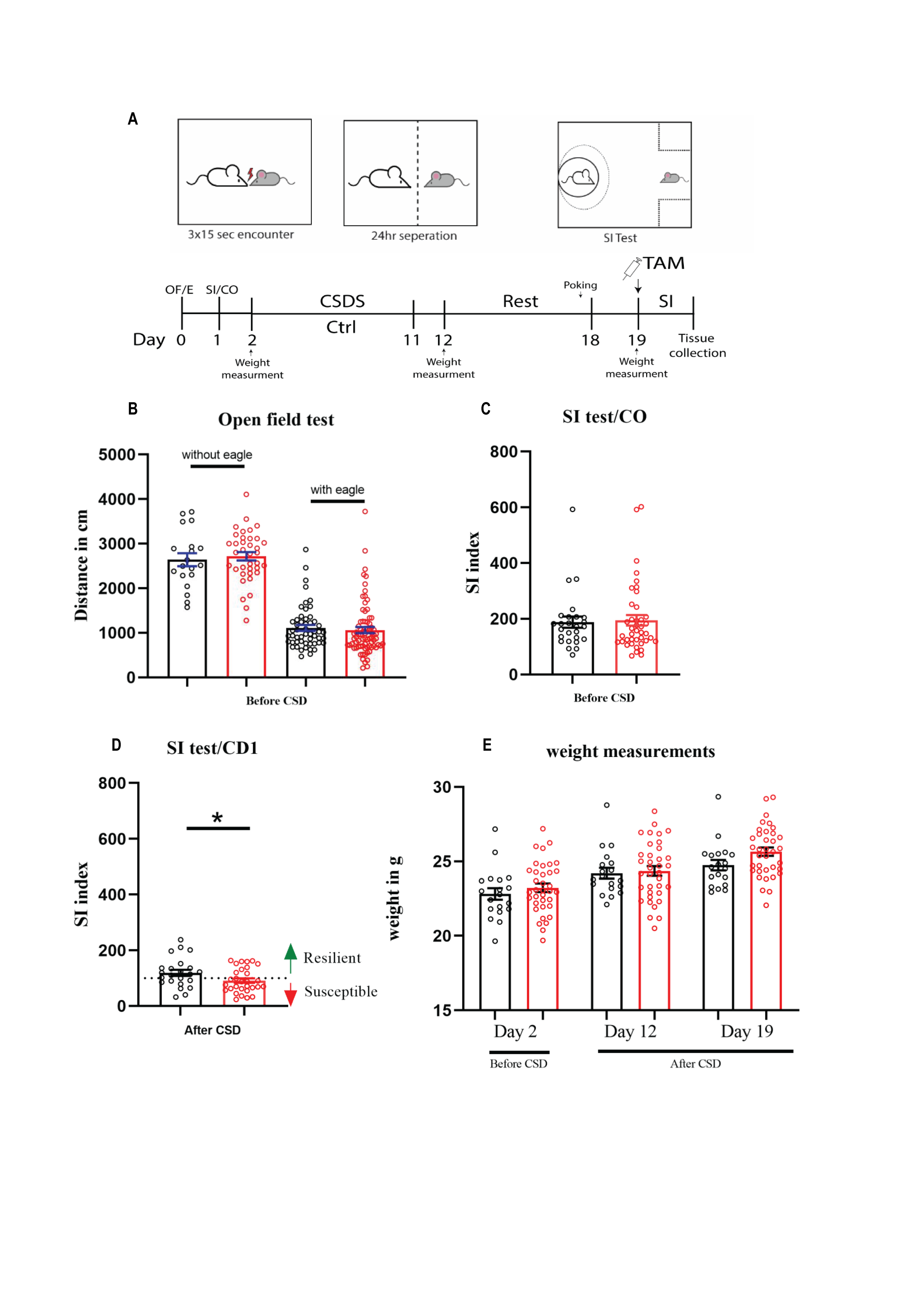
Supplementary Figure 1. Experimental outline for CSDS and results from the WT-GFP.**

A. Principle of CSD. Open field/eagle (OF/E) tests and social interaction test with conspecifics (SI/CO) were performed for a non-biased segregation of test animals to control or stressed groups. CSD was performed for 10 days with 15S attacks repeated 3 times per day. After 7 days of rest, TAM (150 mg/kg) was injected 5 hours before the social interaction test with CD1 to segregate into susceptible and resilient groups. 72 hours after the TAM injection, representative populations of SI-determined resilient, susceptible and control mice were sacrificed. Tissue regions of PFC and vHIP were then isolated from individual mouse. Weights were also measured periodically to assess stress-related modifications as shown in the scheme. B-E. Behaviour assessments of WT-GFP mice: Segregation of the test animals into control (black) and stressed (red) groups, based on their baseline behaviours of open field/eagle exploration (B) or social interactions (C) with conspecifics before CSD, indicating a non-biased segregation of test animals to control or stressed groups. D. Stressed group revealed a significant decrease in social interaction compared to control after CSD (t-test, p<0.05). E. Stressed group revealed an observable though non-significant increased weight compared to control following CSD (t-test, n.s.). All error bars indicate +/- S.E.M. Black circles represent individual non-stressed control mouse, while red circles represent individual stressed mouse.

**
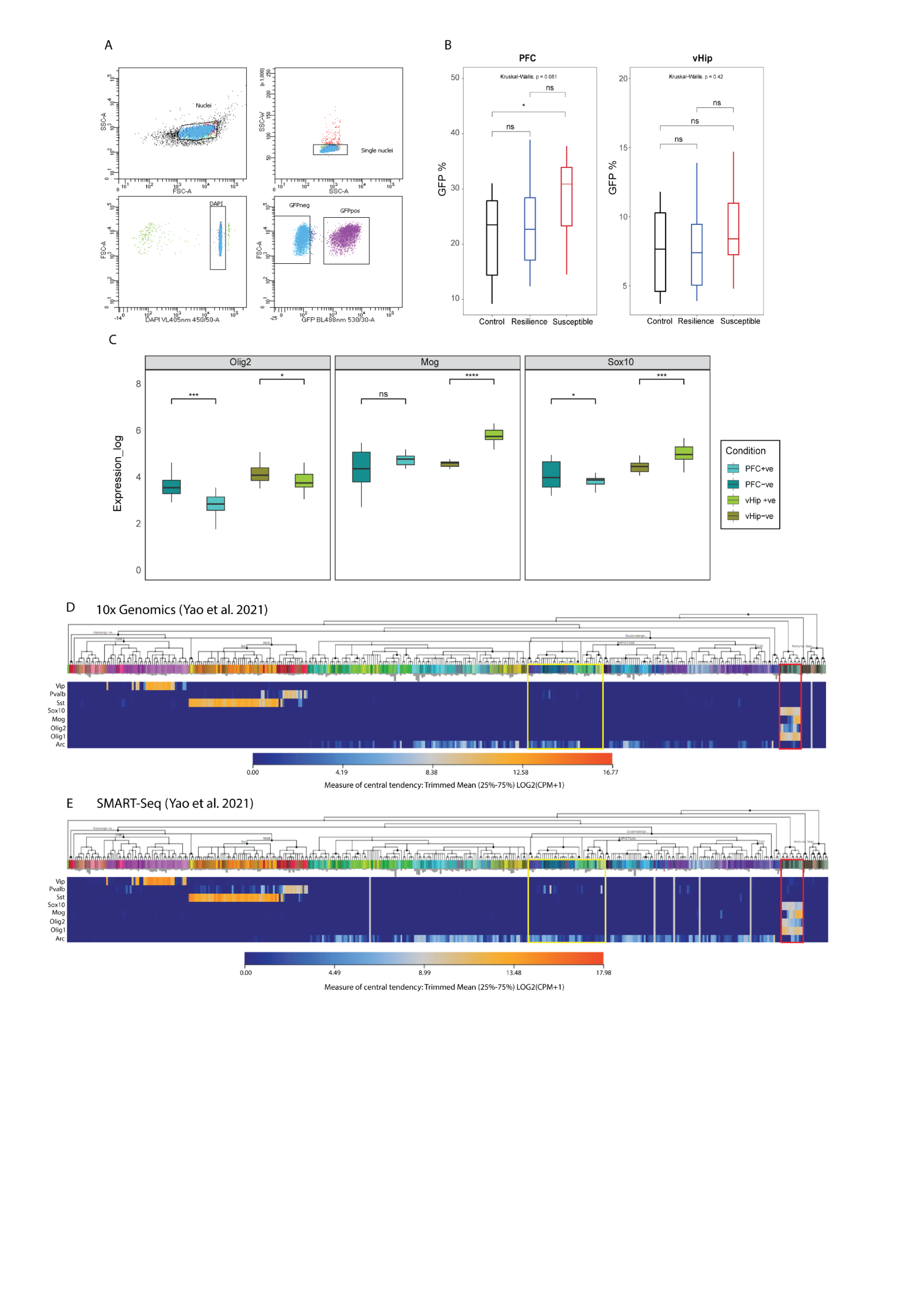
**

**Supplementary Figure 2. Fluorescence activated nuclei sorting and characterization of GFP+ and GFP- nuclei populations.**

A. Following nuclei isolation, nuclei were gated according to their forward-and side-scatter properties (FSC-A/SSC-A) (top left), followed by doublet exclusion using SSC-A and SSC-W (top right). Nuclei were then gated according to their DAPI expression (DAPI VL405nm 450/50-A, bottom left) and GFP expression was used as a sorting gate (GFP BL488nm 530/30-A, bottom right). Sorted nuclei were collected in 1.5 mL Eppendorf tubes, containing the buffers for downstream analyses. B. % GFP expression of resilient, susceptible, and control mice in the PFC and vHIP. Data represents the average of 9-13 biological replicates. (PFC samples n=13,13,9; vHIP data: n=11,12,9; resilience, susceptible and control, respectively, * p<0.05). Error bars represent ±SD. C. Gene expression of glial (*Olig2, Mog, Sox10*) across GFP+ and GFP- samples in the vHIP and PFC. *p<0.05, ***p<0.001, ****p<0.0001. Box plots represent log expression mean. D-E. Single cell level expression of selected markers along with *Arc* as annotated in “Allen Brain Map” database (Yao et al. 2021). The heatmap represents the trimmed mean expression of single cell clusters labelled according to their cell-type in two independent sequencing platforms: 10X Genomics (D) and SMART-Seq (E). Clustered cell-types belonging to L5, L4, L5/6 NP CTX and oligodendrocytes are highlighted in yellow and red, respectively. For additional information, see “Allen Brain Map” database (Yao et al. 2021).

**
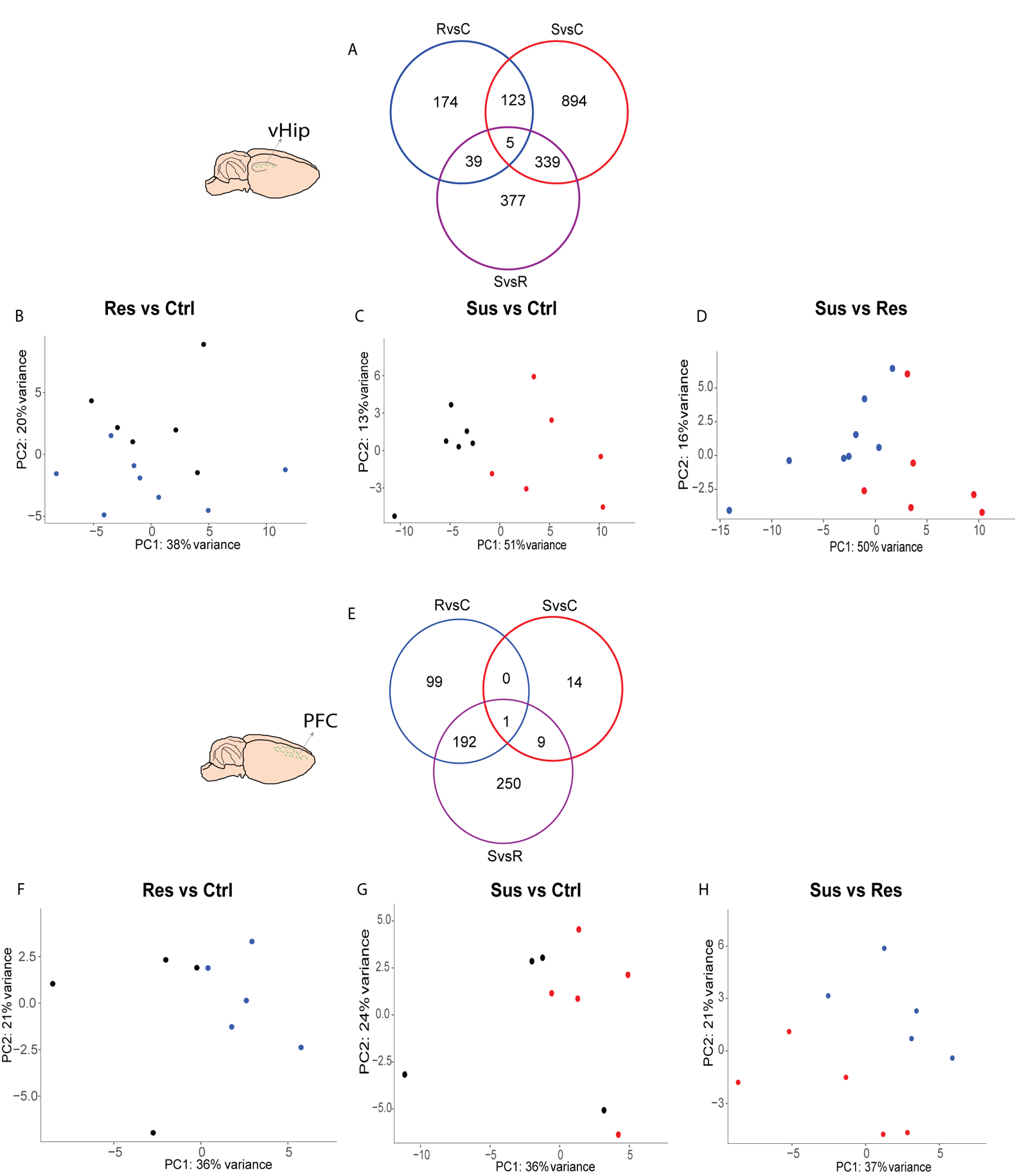
**

**Supplementary Figure 3. Transcriptional analysis across the distinct comparison groups in vHIP and PFC.**

A. Venn diagram presenting the differently expressed genes (DEGs) between resilient vs control (blue), susceptible vs. control (Red) and susceptible vs. resilient (purple) in vHIP. B-D. Principal component clustering analysis (PCA) of Res vs Ctrl (B), Sus vs Ctrl (C) and Sus vs Res (D) in vHIP. Resilience, Ctrl and Susceptible are represented as blue, black and red dots, respectively. E. Venn diagram presenting the differently expressed genes (DEGs) between resilient vs control (blue), susceptible vs. control (Red) and susceptible vs. resilient (purple) in PFC. F-H. Principal component clustering analysis (PCA) of Res vs Ctrl (F), Sus vs Ctrl (G) and Sus vs Res (H) in PFC. Resilience, Ctrl and Susceptible are represented as blue, black and red dots, respectively.

**
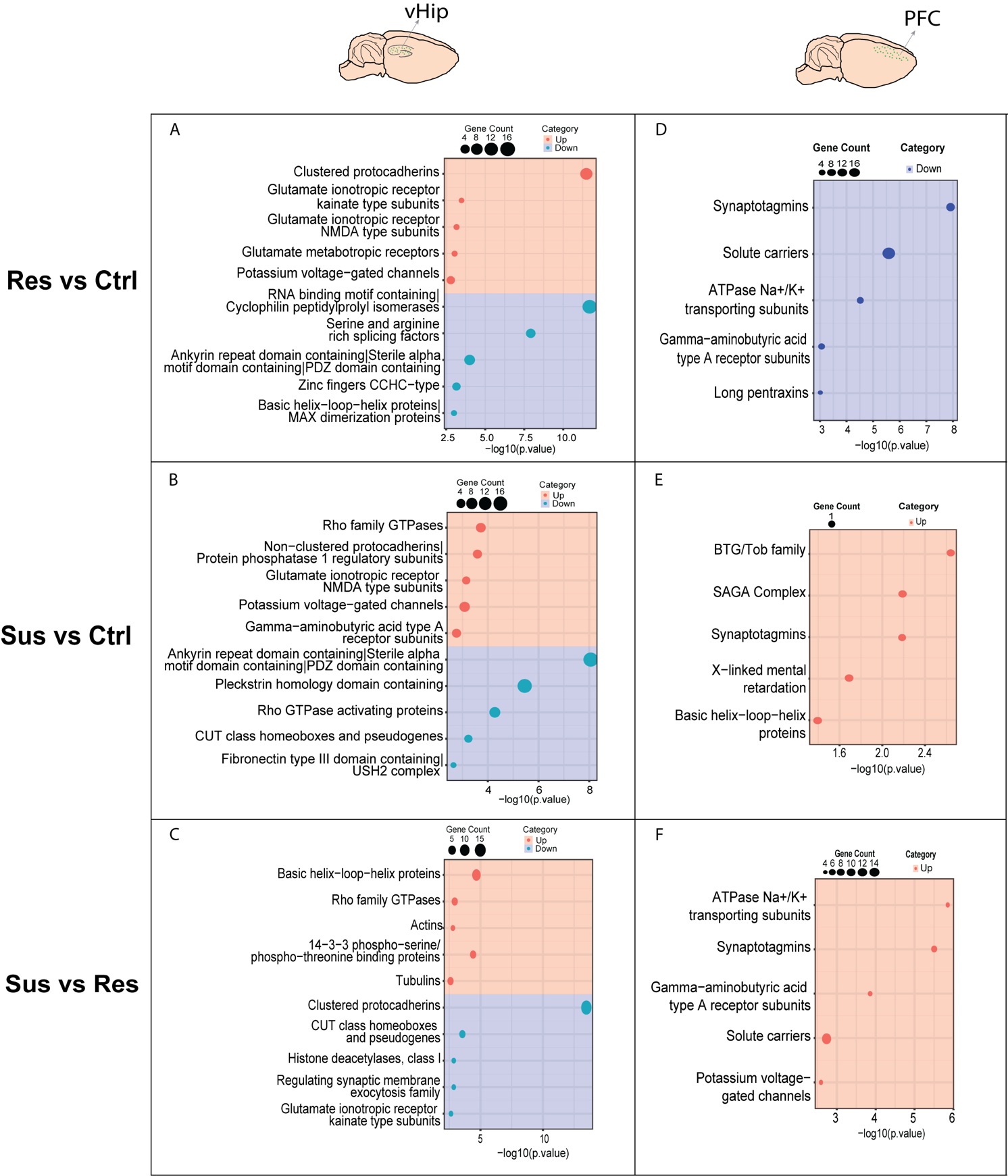
Supplementary Figure 4.** **Characterization of DEGs according to the gene family in the vHIP and PFC**.

A-F. Gene family classification of R vs. C (A, D), S vs. C (B, E) and S vs. R (C, F) in vHIP (A-C) and PFC (D-F).

**
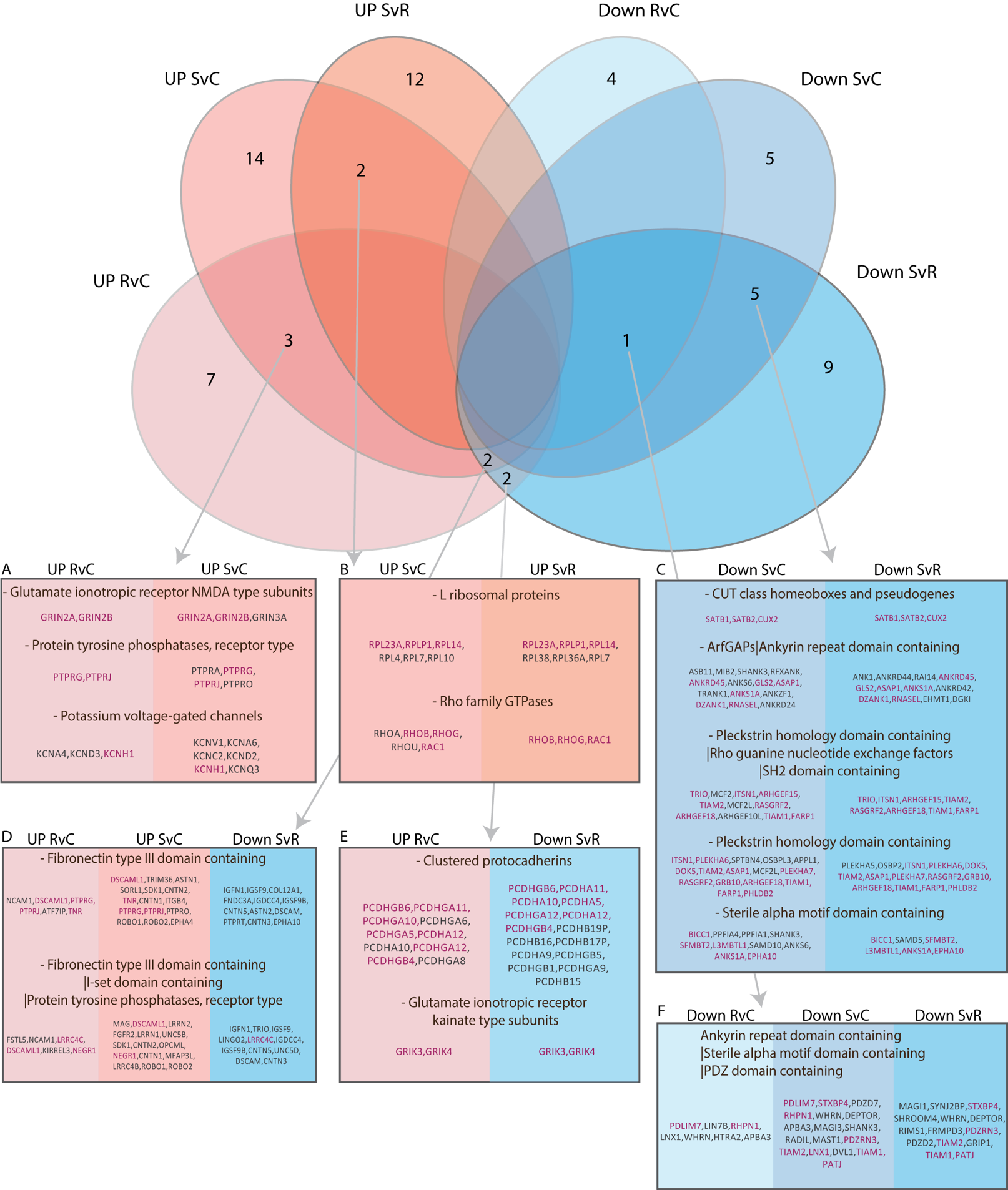
Supplementary Fig.5 Overlap of classified gene families across distinct behavioral groups in vHIP.**

Gene family classification was obtained using ToppGene database and gene families were overlapped across distinct behavioral comparison groups. Each behavioral comparison comprised of DEGs classified according to their gene families (Supplementary Table 4). Gene families were overlapped revealing 6 groups of shared genes families. Genes in purple represent shared DEGs among different comparison groups.

**
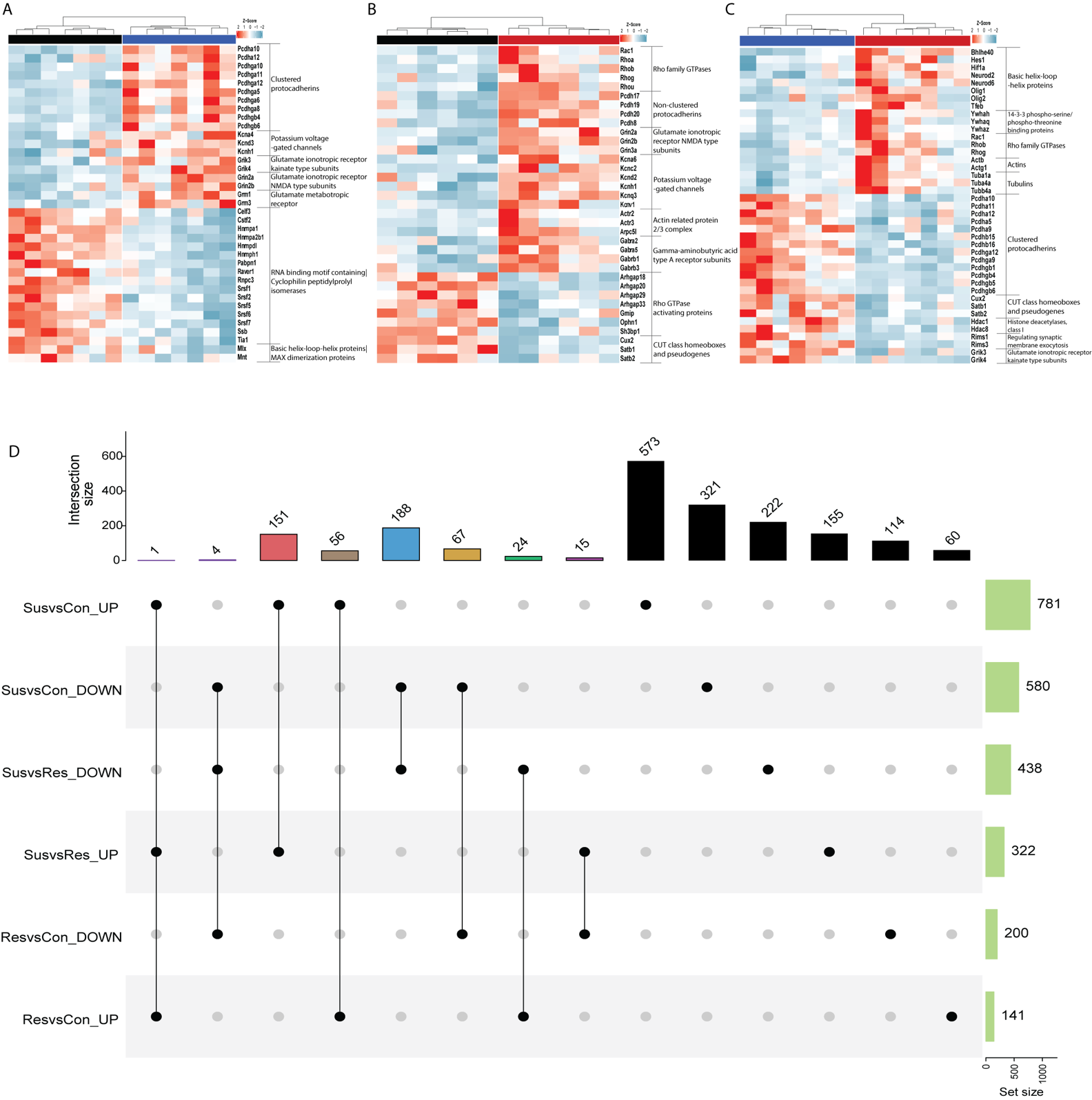
Supplementary Figure 6.** **Selected DEGs classified according to gene families and identification of shared DEGs associated with susceptible and resilient activated nuclei in the vHIP.**

A-C. Heatmaps of selected DEGs classified according to their gene family in Res vs Ctrl (A), Sus vs Ctrl (B) and Sus vs Res (C) (Res – blue, Ctrl – black and Sus – red). Gene expression are represented by expression z-scores computed for selected DEGs. D. Upset plot displaying the overlapping DEGs across the distinct behavioral conditions in vHIP. DEGs were classified as total upregulated in susceptible compared to resilient (red and purple) and total upregulated resilient compared to susceptible (green and blue).

**
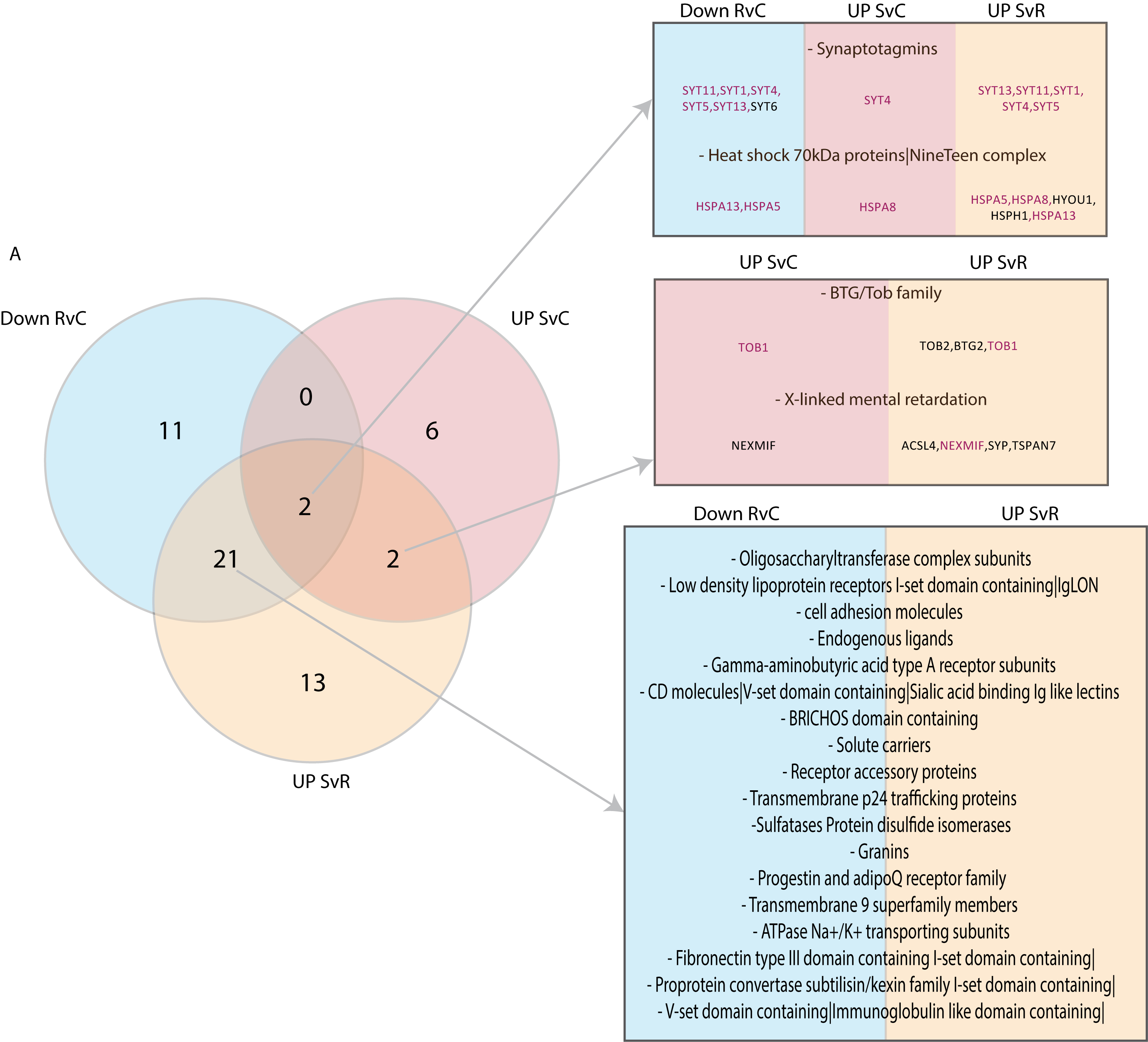
Supplementary Figure 7. Overlap of classified gene families across the distinct behavioral groups in the PFC.**

Gene family classification was obtained using ToppGene database and gene families were overlapped across distinct behavioral comparison groups. Each behavioral comparison comprised of DEGs classified according to their gene families (Supplementary Table 4). Gene families were overlapped revealing 3 groups of shared genes families. Genes in purple represent shared DEGs within different comparison groups.

**
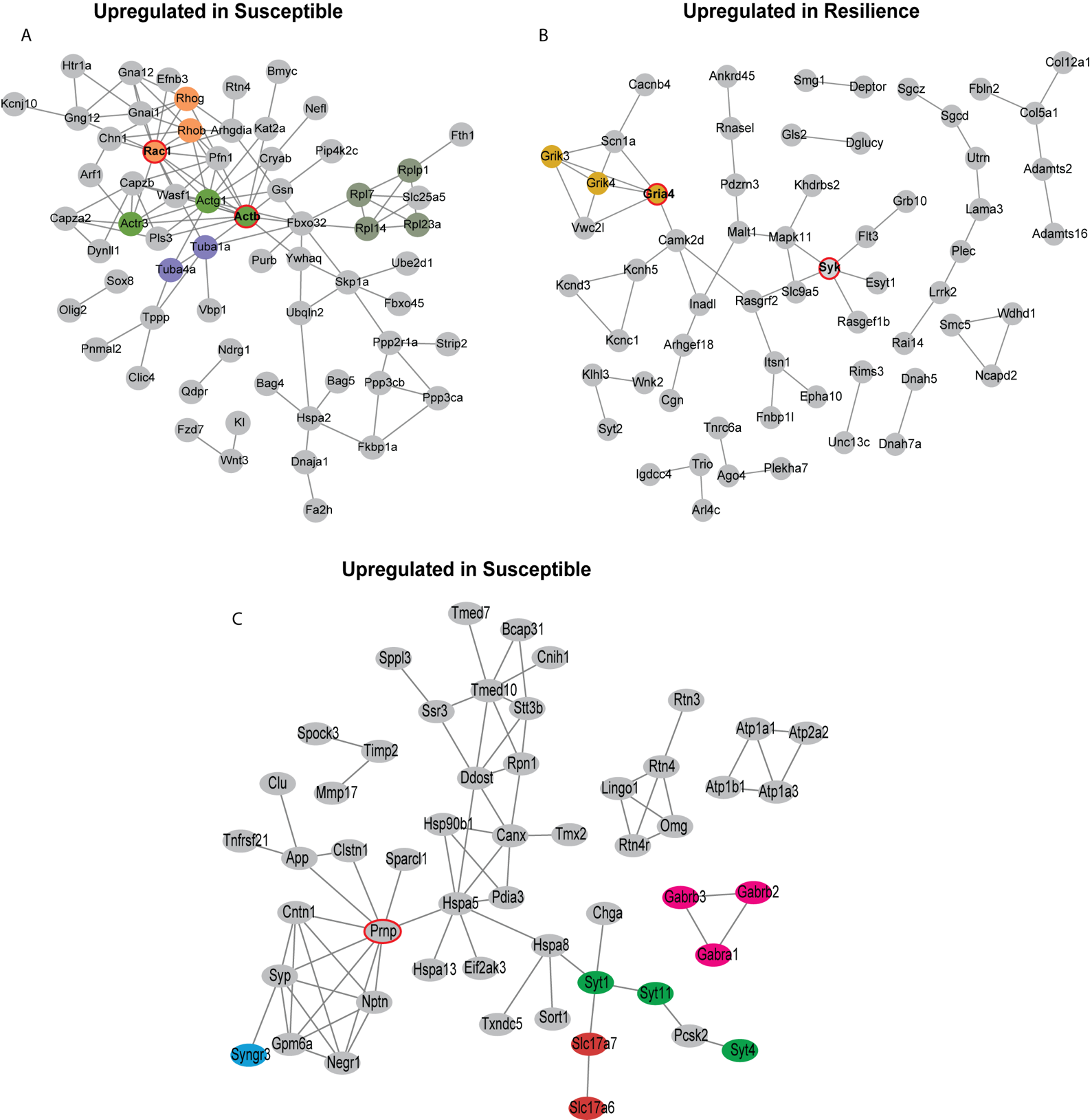
Supplementary Figure 8. Protein-Protein interaction network in vHIP and PFC AN**.

A-B. String network of susceptible specific and resilience specific genes. Circled in red are hub genes predicted by cytohubba. C. String network of susceptible specific genes. Circled in red is hub gene in the network.

**
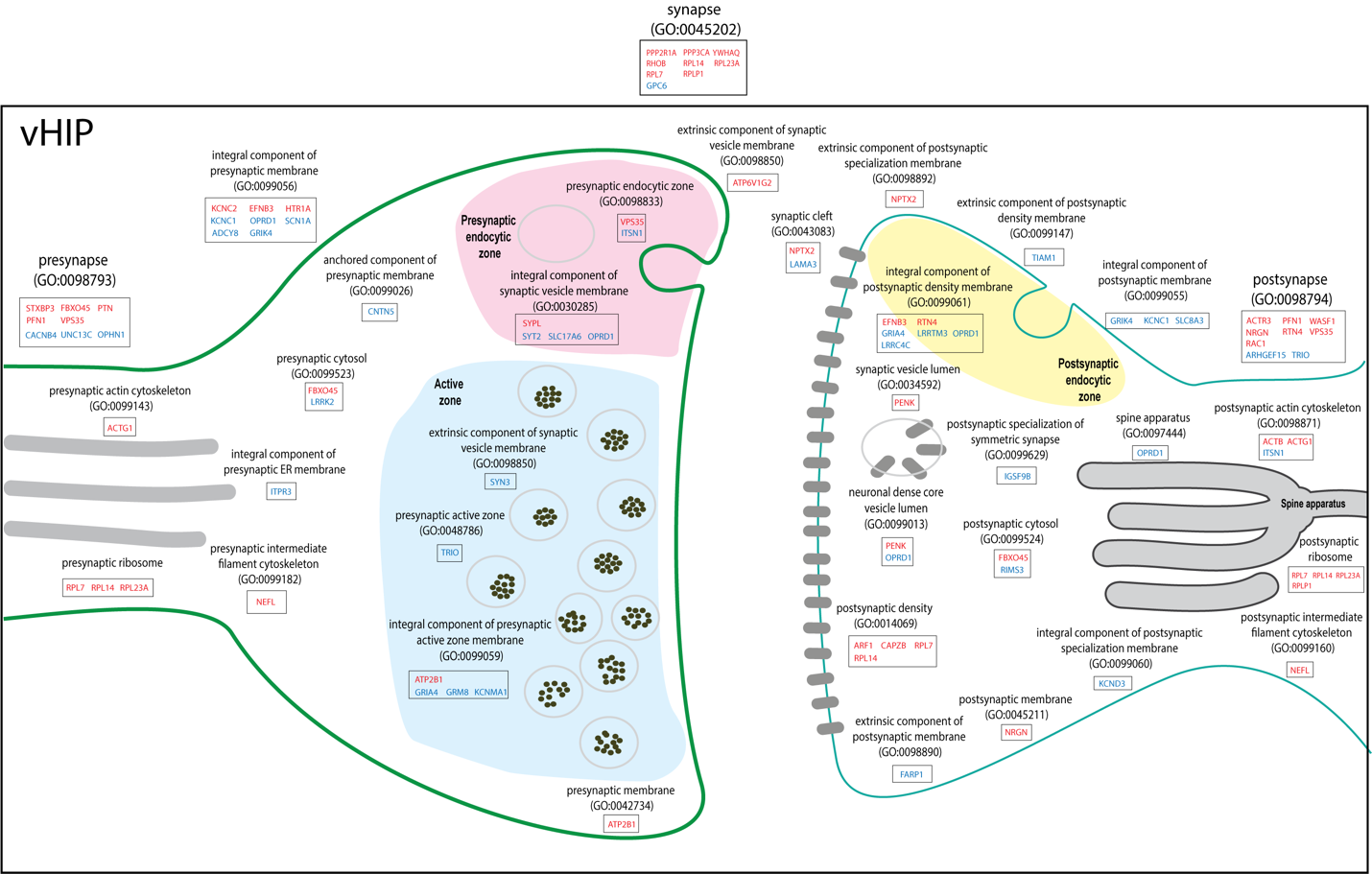
Supplementary Figure 9. Visual representation of DEGs and their corresponding Gene Ontology (GO) terms, as classified by SynGO, within a hypothetical synapse located in the vHIP.**

Upregulated genes in susceptible are highlighted in red while upregulated in resilient are highlighted in blue. Synapse illustration was taken from SwissBioPics database (SIB). The figure provides annotations of DEGs based on the classified SynGO Gene Ontology (GO) terms. Therefore, the annotations were systematically arranged accordingly, yet it is plausible for actual gene functions to manifest in diverse regions within the synapse.

**
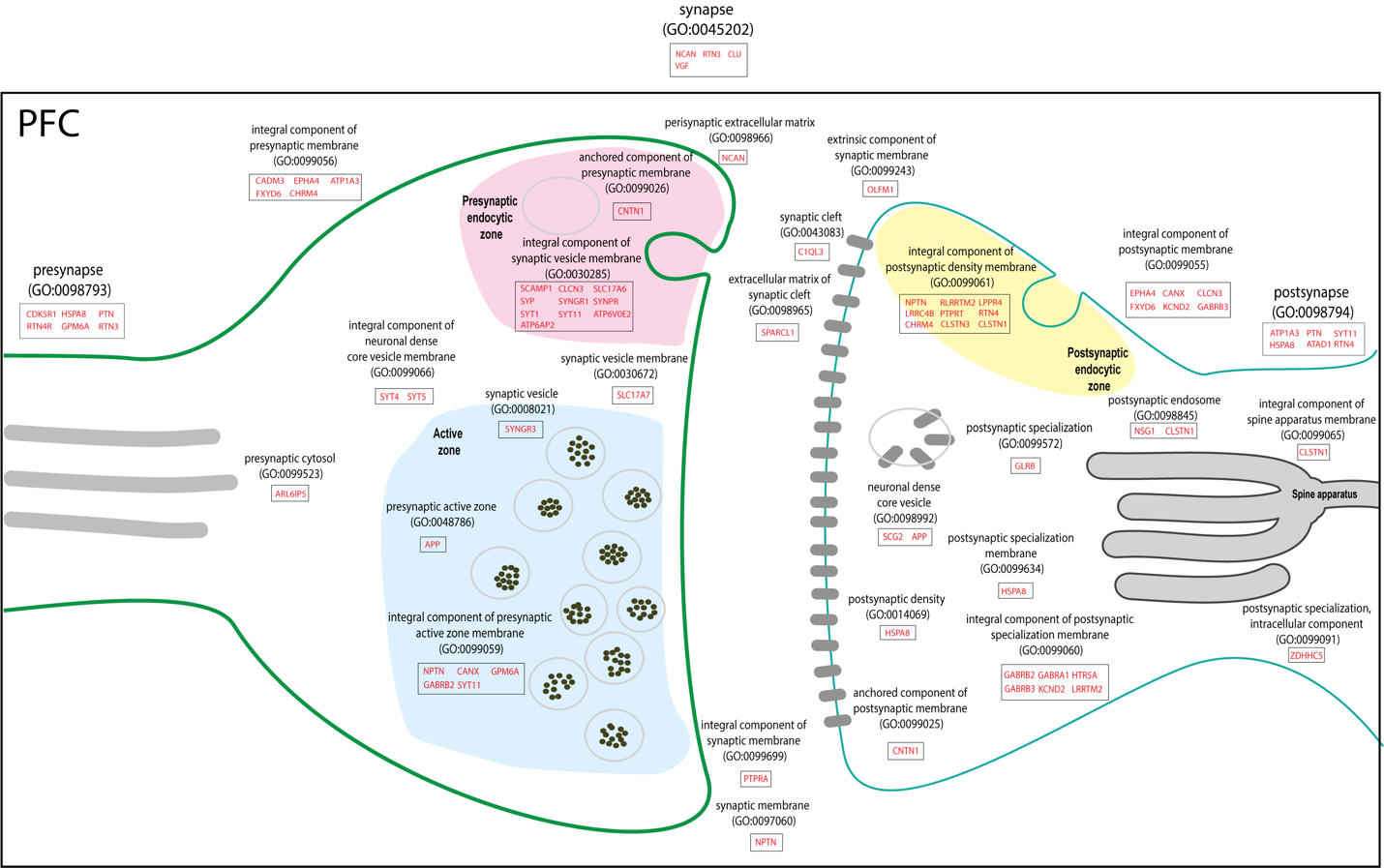
Supplementary Figure 10. Visual representation of DEGs and their corresponding Gene Ontology (GO) terms, as classified by SynGO, within a hypothetical synapse located in the PFC.**

Upregulated genes in susceptible are highlighted in red while upregulated in resilient are highlighted in blue. Synapse illustration was taken from SwissBioPics database (SIB). The figure provides annotations of DEGs based on the classified SynGO Gene Ontology (GO) terms. Therefore, the annotations were systematically arranged accordingly, yet it is plausible for actual gene functions to manifest in diverse regions within the synapse.
